# Quantifying Organic Cation Ratios in Metal Halide Perovskites: Insights from X-ray Photoelectron Spectroscopy and Nuclear Magnetic Resonance Spectroscopy

**DOI:** 10.1021/acs.chemmater.4c00935

**Published:** 2024-07-05

**Authors:** Tatiana Soto-Montero, Suzana Kralj, Jennifer S. Gómez, Jop W. Wolffs, Nathan Rodkey, Arno P. M. Kentgens, Monica Morales-Masis

**Affiliations:** † MESA+ Institute for Nanotechnology, University of Twente, 7500 AE Enschede, The Netherlands; ‡ Institute for Molecules and Materials, Radboud University, 6525 AJ Nijmegen, The Netherlands; § Instituto de Ciencia Molecular, Universidad de Valencia, 46980 Paterna, Spain

## Abstract

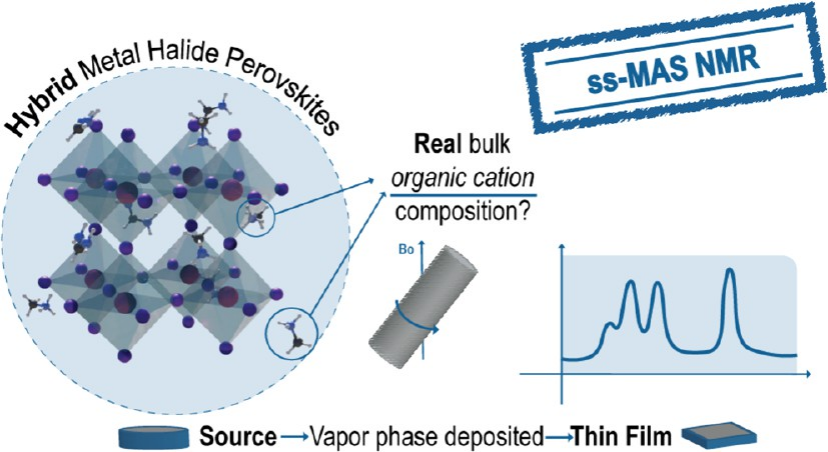

The employment of metal halide perovskites (MHPs) in various optoelectronic applications requires the preparation of thin films whose composition plays a crucial role. Yet, the composition of the MHP films is rarely reported in the literature, partly because quantifying the actual organic cation composition cannot be done with conventional characterization methods. For MHPs, NMR has gained popularity, but for films, tedious processes like scratching several films are needed. Here, we use mechanochemical synthesis of MA_1–*x*_FA_*x*_PbI_3_ powders with various MA^+^: FA^+^ ratios and combine solid-state NMR spectroscopy (ssNMR) and X-ray photoelectron spectroscopy (XPS) to provide a reference characterization protocol for the organic cations’ quantification in either powder form or films. Following this, we demonstrate that organic cation ratio quantification on thin films with ssNMR can be done without scraping the film and using significantly less mass than typically needed, that is, employing a single ∼800 nm-thick MA_1–*x*_FA_*x*_PbI_3_ film deposited by pulsed laser deposition (PLD) onto a 1 × 1 in.^2^, 0.2 mm-thick quartz substrate. While background signals from the quartz substrate appear in the ^1^H ssNMR spectra, the MA^+^ and FA^+^ signals are easily distinguishable and can be quantified. This study highlights the importance of calibrating and quantifying the source and the thin film organic cation ratio, as key for future optimization and scalability of physical vapor deposition processes.

## 1 Introduction

Metal halide perovskites (MHPs) are highly versatile materials with tunable bandgaps that can be stabilized in the photoactive phase through using multiple cations or halide mixtures.^1−3^ However, accurately determining the composition of these complex compounds, mainly those containing organic and inorganic components, presents a significant challenge.^4,5^ In fact, the actual composition of hybrid MHP thin films is rarely reported in the literature.^6^ Instead, the nominal precursor ratios for solution-based processes^7^ or deposition rates determined by the quartz crystal monitors during coevaporation^8^ are more commonly stated. This is mainly due to the fact that typical bulk-sensitive chemical compositional techniques such as energy-dispersive X-ray spectroscopy (EDX), Rutherford backscattering spectroscopy (RBS), and X-ray fluorescence spectroscopy (XRF) do not provide information on the organic species as they are insensitive to light atoms such as C, H, or N.

For the typically used organic components, methylammonium (MA^+^ = CH_3_NH_3_^+^) and formamidinium (FA^+^ = CH(NH_2_)_2_^+^), solid-state NMR spectroscopy (ssNMR)^9−11^ has emerged as a powerful technique giving important insights into the local environment of these organic molecules via ^1^H, ^13^C, ^15^N, or ^14^N analysis.^9^ Other NMR-active nuclei can be deployed in current MHP materials research (^35^Cl, ^39^K, ^79^Br, ^87^Rb, ^119^Sn, ^127^I, ^133^Cs, and ^207^Pb), each nucleus requiring distinct acquisition strategies.^9,12^ Contrary to diffraction-based methods, ssNMR can detect and quantify secondary phases, amorphous components, or partly disordered species.^13,14^ Applying ssNMR for MHP research can provide valuable and even unique information into dopant incorporation,^15^ cation dynamics,^11,16−20^ compositional quantification,^21,22^ phase segregation,^23^ halide mixing,^10,18,24,25^ degradation pathways,^21,26,27^ surface passivation mechanisms,^28,29^ surface termination,^30^ precursor quality,^25,31−33^ and other critical aspects.^9^

Although the main aspects of ssNMR materials research and experimental details have been recently reviewed^9,12−14^ and ssNMR is increasingly employed in MHPs quantum dots,^34^ powders,^18^ and films,^35^ challenges persist when it comes to the analysis of thin films due to limited sample yield, that is, usually needing >500 nm-thick perovskites or multiple thin films, followed by scraping the film from the substrate.^28,35,36^ An alternative approach, proposed by Hanrahan et al.,^37^ involves crushing the thin film substrate into small pieces using a mortar and pestle and subsequently packing them into an NMR rotor. Using this approach, the authors demonstrated the acquisition of fast MAS ^207^Pb NMR spectra of intact MAPbI_3_ films and that such analysis could be transferred to more complex MHP compositions to elucidate and quantify the distinct lead coordination environments. In addition, the advent of solid-state mechanochemical synthesis for MHPs has helped the adoption of ssNMR to analyze these materials as well.^16,38,39^ Mechanochemical synthesis has proven to be a highly effective method for providing large quantities of high-purity and crystalline MHP materials of the desired composition without solvent interference, allowing for highly sensitive ssNMR studies including MAPbI_3_, FAPbI_3_, MA_1–*x*_FA_*x*_PbI_3_, Cs_1–*x*_FA_*x*_PbI_3_, (RbCsFAMA)Pb(I_*y*_Br_1–*y*_)_3_, CsFAMAPb(I_*y*_Br_1–*y*_)_3_, and MAPbI_*y*_Br_1–*y*_.^40−44^ Overall, NMR spectroscopy stands out as a versatile method for quantitative characterization of MHPs and the entire system they are a part of, including solvents, additives, phase composition, etc., resulting from the synthesis procedure.^25,45^ Besides structural information, solid-state NMR techniques offer valuable insights into the local dynamics and the interactions that impact those dynamics. As such, it can relate properties at the atomic level to the final properties of the thin film or even the optoelectronic device.

X-ray photoelectron spectroscopy (XPS) provides complementary information regarding the chemical environment; for example, by studying the N 1s core levels, the ratio of MA^+^ and FA^+^ cations in hybrid MHPs can be accurately quantified after proper calibration. This calibration requires consideration of the system geometry and sensitivity factor of the elements. XPS is a surface-sensitive technique widely employed in the study of the surface chemistry of mixed MHPs thin films, particularly for analyzing surface termination and the effects of surface post-treatment.^28,46−50^ Nevertheless, only a few studies have investigated bulk MHP materials using XPS.^51−53^

The most efficient perovskite solar cells today are based on complex A-site cation and halide mixtures.^54,55^ A precise determination of cation ratios in mixed MHP compositions is therefore relevant for transferring results between laboratories and future scalability efforts with different fabrication methods. For example, single-source vapor deposition methods, including single-source evaporation, flash evaporation, sputtering, and pulsed laser deposition (PLD),^56^ are attractive due to the reduced hardware complexity. However, these methods typically utilized presynthesized MHP as a single source material for thin film growth. Previously, we explored PLD as a single-source physical vapor deposition (PVD) technique to grow MHP containing double organic cations, making clear the importance of controlling the single-source organic cation ratio to obtain the desired thin film stoichiometry.^35^

Motivated by this, in this work, we first use all-dry mechanochemical synthesis via ball-milling to fabricate mixed-cation MA_1–*x*_FA_*x*_PbI_3_ (*x* = 0–1) hybrid halide perovskites and directly compare their characterization via ssNMR and XPS spectroscopy. This characterization is complemented with structural and optical properties analysis via specular X-ray diffraction (XRD) and photoluminescence (PL). These measurements then serve as a calibration to determine the actual cation ratio during the fabrication of vapor deposition sources, which later on could be used for the growth of MA_1–*x*_FA_*x*_PbI_3_ thin films. Subsequently, we explore the analysis of a single ∼800 nm MA_1–*x*_FA_*x*_PbI_3_ (*x* ∼ 0.61) thin film grown via PLD on a 0.2 mm thick quartz substrate (1 × 1 in.) using ^1^H solid-state NMR to determine the cation ratio quantitatively.^11,16,57^ The presence of hydrogen in both cations makes this quantification possible with a single spectrum, but it should be noted that this approach can be extended to systems that have inorganic cations mixed in by performing quantitative measurements relating to the measurement of a reference compound with a known amount of protons. Similarly, this would be possible by such measurements of the inorganic cation if its receptivity is high enough. Furthermore, these measurements reveal a noteworthy advancement in ssNMR of perovskite thin films, as only one thin film 800 nm thick is required instead of multiple films for quantifying the cation ratio by ssNMR. The outcome of this study has important implications for advancing our comprehension of the impact of perovskite precursors on the final thin films’ actual composition that can vary significantly between deposition methods and perovskite compositions.

## II Experimental Methods

All starting precursor materials were obtained from commercial sources and used without further purification: methylammonium iodide (MAI, >99.99%, Greatcellsolar), formamidinium iodide (FAI, >99.99%, Greatcellsolar), and lead iodide (PbI_2_, 99.999% trace metals basis, perovskite grade, Sigma-Aldrich).

### II.I Mechanochemical Synthesis of MA_1–*x*_FA_*x*_PbI_3_ (*x*: 0–1)

To prepare MA_1–*x*_FA_*x*_PbI_3_ with x ranging from 0 to 1, stoichiometric molar ratios of powder precursors were mixed in zirconia jars under an N_2_ atmosphere. The resulting mixture was ball milled at room temperature (RT) for 48 h using zirconia balls with a fixed ball-to-powder ratio (BPR) of 10. The molar ratio of organic components MAI:FAI was varied from 1:0 (MAPbI_3_) to 0:1 (FAPbI_3_), with intermediate targeted compositions of MA_0.75_FA_0.25_PbI_3_, MA_0.63_FA_0.37_PbI_3_, MA_0.50_FA_0.50_PbI_3_, and MA_0.25_FA_0.75_PbI_3_. Approximately 1.0 g of each powder composition was reserved for ssNMR measurements, while ∼2.0 g was uniaxially pressed at RT for 30 min at 158 MPa into a disc-shaped pressed powder (dimensions: 1.3–1.5 mm thick and 20 mm in diameter). Further details can be found in the Appendix Tables S8–S15.

### II.II Powder X-ray Diffraction (XRD) Measurements

The pressed mechanochemically synthesized (MCS) powders were measured in air using a PANalytical X’Pert PRO system with a Cu anode X-ray source with a symmetric configuration. Temperature-dependent XRD measurements were conducted using an MRD XRD system with an Anton Paar DHS900 heating stage.

### II.III Photoluminescence (PL) Measurements

Steady-state PL measurements were carried out using a custom-built setup comprising a 520 nm laser diode module with a power output of 100 mW (Matchbox series) and a StellarNetBLUE-Wave spectrometer coupled with a fiber optic cable.

### II.IV X-ray Photoelectron Spectroscopy (XPS)

Surface chemical environment analysis of the pressed MCS powders was performed using an Omicron XM 1000 Al-Kα monochromated X-ray source (1486.6 Ev, fwhm = 0.26 Ev) and an Omicron EA 125 energy analyzer with a pass energy of 50 eV, at a photoemission angle θ of 55°. An electron neutralizer beam is used to minimize binding energy shifts. The measurements were carried out at a pressure of <3 × 10^–10^ mbar. The samples were affixed with a copper double-sided conductive adhesive tape and analyzed as loaded. The peak positions and width were fitted using a Gaussian–Lorentzian function (GL) with Shirley background via CasaXPS software. The peak position and peak area constraints were applied during fitting based on reported data. Adventitious carbon was set at 284.85 eV for all samples. The cation ratios were qualitatively analyzed for each pressed powder through the N 1s core-level analysis (quantitative analysis requires correction for the system geometry and SF). The CasaXPS software was employed for peak fitting and quantification, and from these results, the error bars were also extracted.

### II.V Magic Angle Spinning Solid-State NMR (MAS ssNMR)

A set of five samples were used for NMR analysis: four pressed MCS powders (MA_1–*x*_FA_*x*_PbI_3_ with *x* = 0.25, 0.37, 0.50, and 0.75) and one MA_1–*x*_FA_*x*_PbI_3_ (with *x* = 0.25 from PLD source target) thin film of approximately 800 nm thick, which was coated on a 0.2 mm thick quartz substrate (100) of 1 × 1 in. using pulsed laser deposition (PLD). The thin film was crushed in air using a mortar and pestle. For all solid-state NMR experiments, rotors were packed in air. Note: an 800 nm-thick film was chosen to enhance the signal-to-noise (S/N) ratio in this initial experiment (the substrate occupies 99.6% of the volume of the rotor). The quartz substrates were sonicated in acetone, isopropanol, and water for 10 min each and used without further treatments.

All experiments on the perovskite powders were recorded with a Bruker Avance NEO 600 MHz spectrometer (*B*_0_ = 14.09 T, *ν*_0_ = 600.13 MHz for ^1^H). A 3.2 mm HXY MAS Varian probe operating in double resonance mode was used. The samples were spun at *ν*_R_ = 12.5 kHz. Quantitative ^1^H NMR spectra were acquired using single-pulse excitation (SPE) NMR experiments with a pulse length of 2.17 μs, averaging 16 transients separated by a recycle delay of ≥5*T_1_ = 80–130 s. Background suppression was achieved by subtracting an analogous acquisition of the empty rotor. ^1^H *T*_1_ relaxation of each sample was determined by using the saturation recovery method. ^13^C NMR spectra were acquired by using SPE NMR experiments with a pulse length of 4 μs corresponding to an rf field of *ν*_1_ = 63 kHz, 64 transients, a recycle delay of ≥5*T_1_ = 200–280 s, and empty rotor subtractions. The ^13^C *T*_1_ relaxation of each sample was measured using the inversion recovery method. ^13^C CPMAS experiments were acquired under the following experimental conditions: ^1^H 90° pulse was set to 2.17 μs corresponding to an rf-field of ∼115 kHz. A VACP contact time of 10 ms using a 50–100% ramp at the ^1^H channel was employed, while the RF nutation frequency on the ^13^C channel was 63 kHz. SPINAL ^1^H decoupling (52 kHz) was applied during acquisition. 64 transients were accumulated, separated by a recycle delay of 80–130 s. All experiments on the perovskite thin film were recorded at *B*_0_ = 19.97 T (*ν*_0_ = 850.13 MHz for ^1^H) on a Bruker Avance NEO spectrometer equipped with a 1.6 mm triple resonance HXY Varian probe spinning at *ν*_R_ = 25 kHz. Quantitative ^1^H NMR spectra were acquired by using SPE NMR experiments with a pulse length of 2.5 μs. 64 transients separated by a recycle interval of ≥5*T_1_ = 55 s were acquired. Again, the background was removed by subtracting the spectrum of an empty rotor acquired under identical conditions. Chemical shifts for ^1^H and ^13^C were referenced using a solid sample of adamantane as a secondary reference (^1^H δ_iso_ = 1.82 ppm and ^13^C δ_iso_ = 29.47 and 38.52 ppm). All *T*_1_’s can be found in Table S1. Data processing was carried out in Topspin for the pressed powders and ssNake (version 1.4)^58^ for the thin film spectra. Pressed powder spectra had their baselines corrected and were apodized. For the thin film, the first 80 μs of data points was deleted and back predicted. Fitting was done using Lorentzian/Gaussian functions for both pressed powders (both ssNake and Dmfit^59^ to get error estimates) and the thin film (ssNake only).

### II.VI Pulsed Laser Deposition (PLD) of MA_1–*x*_FA_*x*_PbI_3_ Thin Film

A coherent KrF excimer laser (λ = 248 nm) was used to ablate the solid target ([MAI: FAI]: PbI_2_, 8·[0.75:0.25]:1; that is, a target with 0.75 mol of MAI vs 0.25 mol of FAI where the sum of organic moles is 8-fold with respect to the inorganics) inside a customized (TSST Demcon) vacuum chamber, background pressure of 2.0 × 10^–7^ mbar, and working pressure of 0.02 mbar under an argon atmosphere. The depositions were performed at a target-to-substrate distance of 55 mm, with a laser fluence of 0.31 J cm^–2^, and a spot size (target ablated area per pulse) of 2.33 mm^2^. The local frequency was set to 4 Hz for 20000 pulses while scanning a 36 × 36 mm^2^ area at a holder set point temperature of approximately 35 °C on a quartz substrate (SiO_2_, (101̅0) edge (0001) parallel to the long edge, 1 × 1 in., 0.2 mm thick, surface 1 side epi pol; SurfaceNet). Note: here, we demonstrate that 0.2 mm-thick quartz substrates are useful for measurements, but thinner substrates with a partial device stack (glass/ITO/HTL or ETL) are desirable.

## III Results and Discussion

### III.I From Powder to PVD Targets

Mechanochemical synthesis has been used to synthesize MHP powders that were later used for the fabrication of MHP films via solution^41,43,60^ or different vapor-phase growth methods.^61−63^ Among single-source PVD methods, pulsed laser deposition (PLD)^35,64−66^ and sputtering deposition^67,68^ utilize solid targets consisting of mixtures of the MHP precursors. Previously, we demonstrated the critical role of the MHP target composition (including an 8-fold excess of organic cations compared to inorganic components) in determining the final film’s stoichiometry and optoelectronic properties during PLD.^35,69^ Therefore, a precise compositional characterization of both targets and thin films is essential for enhancing the reproducibility of PVD film fabrication.

**Figure 1:**
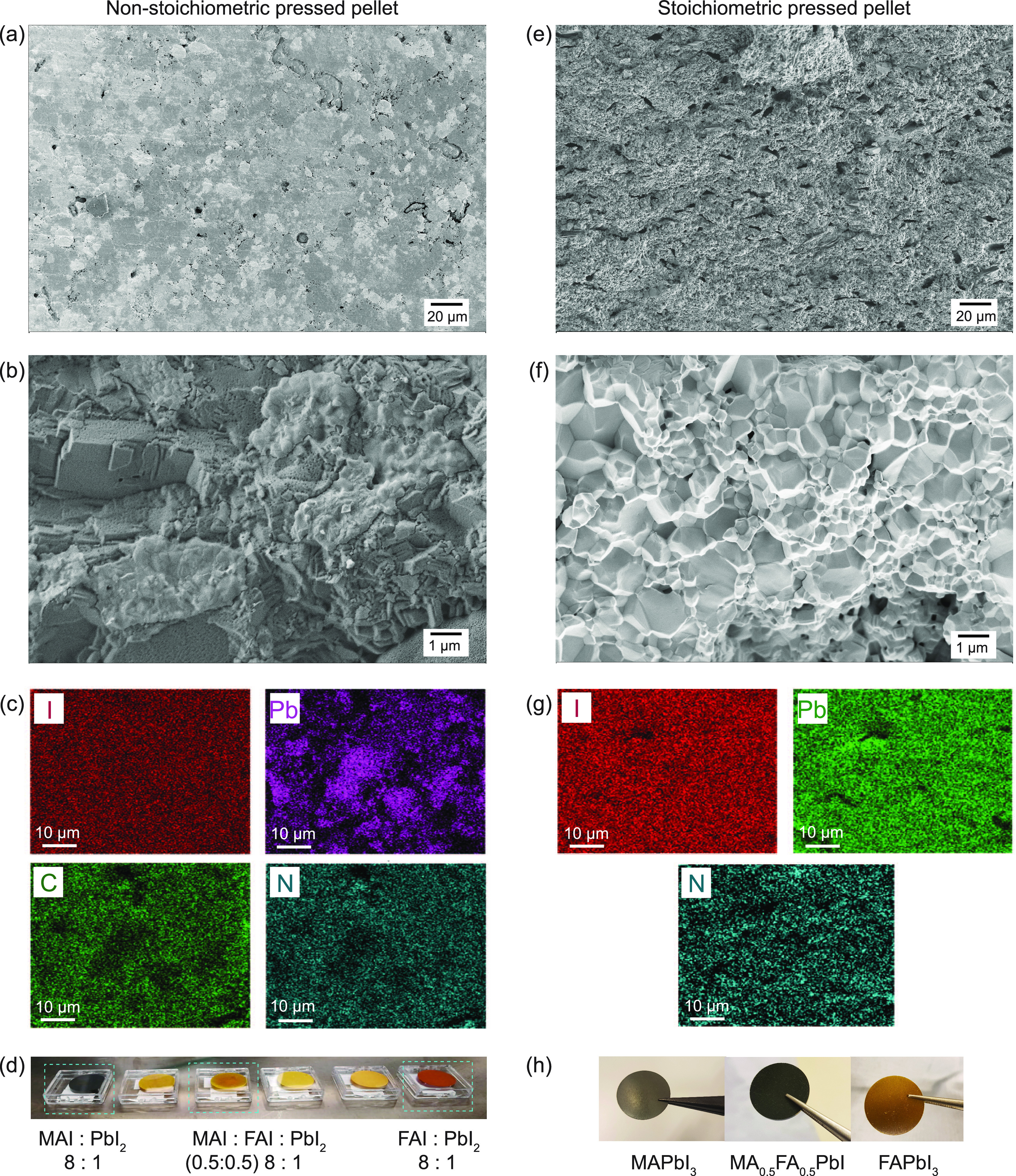
Comparison between a nonstoichiometry pressed pellet (MA_0.75_FA_0.25_)I:PbI_2_ = 8:1 (a–d) and a stoichiometric pressed pellet of MAPbI_3_ (e–h); (a) and (e) SEM top view, (b) and (f) cross section, (c) and (g) EDX maps, and (d) photos of the nonstoichiometric pressed pellets containing different cation ratios MA:FA and 8-fold organics vs inorganic components used as PVD targets and (h) photographs of stoichiometric pressed pellets.

Figure 1 illustrates the morphology of a PVD target fabricated with an 8-fold excess of (MA^+^ and FA^+^) relative to PbI_2_, along with a stoichiometric pressed powder reference. The powders are all prepared via MCS and pressed as described in the Experimental Methods. Notably, the microstructure of both is dense, with visible μm-size crystalline grains only for the stoichiometric pellet. This is expected, as the excess of organics in the nonstoichiometric target might prevent the formation of large crystalline grains. The EDX analysis (Figure 1c,g) reveals a uniform distribution of elements in both cases. Yet, the quantitative assessment of the ratio and integrity of organic compounds is not possible with conventional EDX alone. Therefore, for a more accurate analysis of the PVD target composition, we proceeded to employ techniques such as ssNMR and XPS.

### III.II Compositional Analysis of MCS MA_1–*x*_FA*_x_*PbI_3_

**Figure 2:**
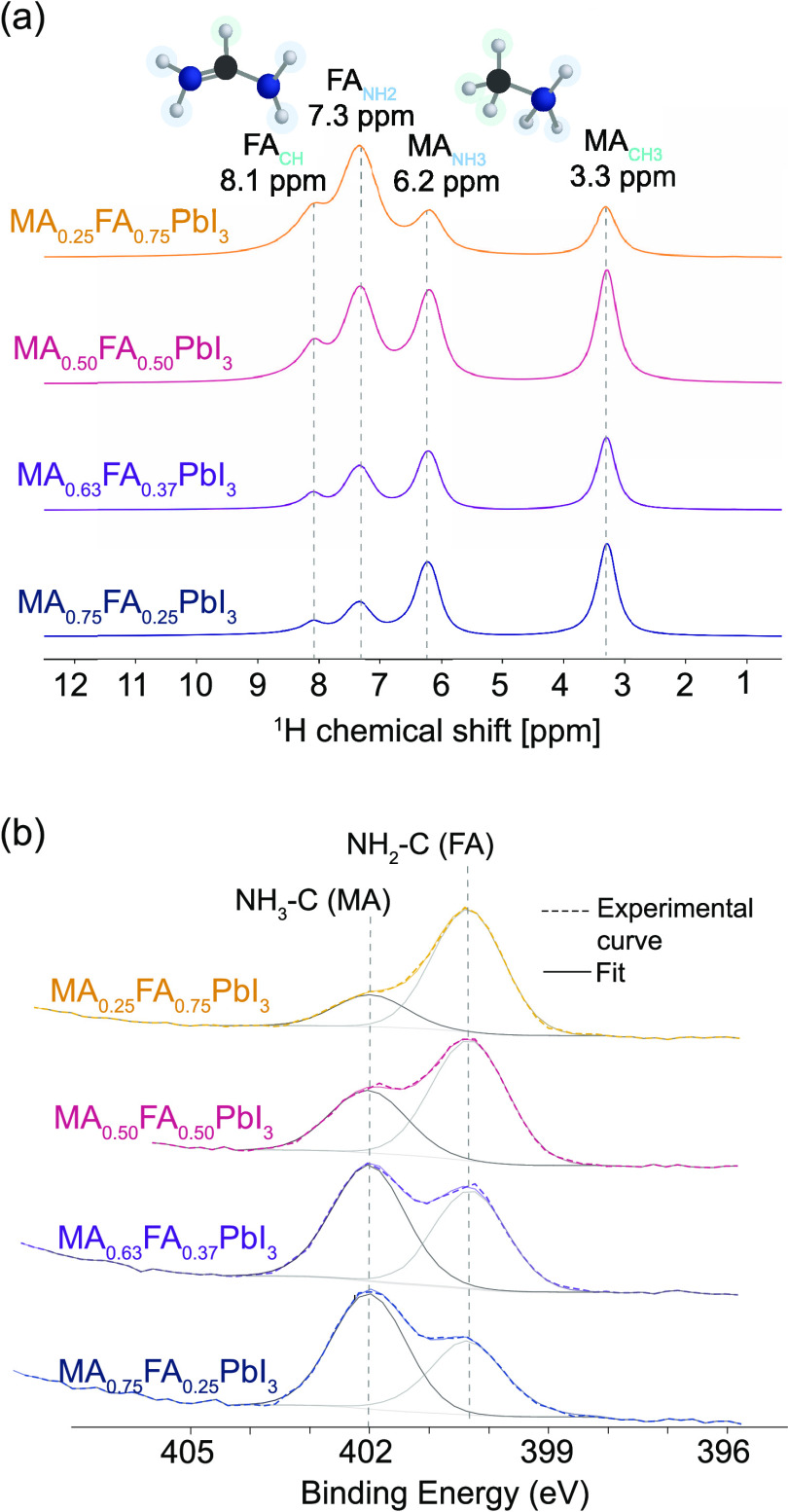
(a) ^1^H MAS NMR quantitative spectra and (b) XPS spectra of N 1s core levels corresponding to the FA^+^ and MA^+^ organic cations obtained for mechanochemically synthesized MA_1–*x*_FA_*x*_PbI_3_ powders with nominal compositions of *x* = 0.25 (shown in blue), *x* = 0.37 (shown in purple), *x* = 0.50 (shown in pink), and *x* = 0.75 (shown in yellow).

Figure 2a displays the ^1^H MAS NMR spectra of four different cation-mixed MA_1–*x*_FA_*x*_PbI_3_ (*x*: 0.25–0.75) powders exhibiting distinct signals assigned to the ^1^H species of the MA^+^ and FA^+^ cations. The chemical shifts at 3.3 and 6.2 ppm correspond to the C**H**_**3**_ group and the N**H**_**3**_ group of the MA^+^ cation, respectively, while the signals arising at 7.3 and 8.1 ppm are in agreement with those of the N**H**_**2**_ groups and the C**H** group of the FA^+^ cation.^13,16,57^

Complementary information from ^13^C MAS NMR experiments of these samples (Figure S1) further reveals the distinctive peaks corresponding to the **C**H_3_ group of the MA^+^ cation at 31.3 ppm and the **C**H group of FA^+^ at ∼156 ppm. Notably, a shoulder for the compositions containing 50% and 75% FA^+^, respectively, is observed. This kind of asymmetrical peak shape has been previously identified for similar mixed-cation perovskites and could indicate the presence of a secondary δ-phase.^11^ Other studies observed degradation toward this yellow δ-phase for double cation compositions with an FA% of ≥80 unless other small cations, such as Cs^+^, are incorporated, even when handled in dry and dark conditions.^2,11,70^ Humidity can also influence this degradation process.^71^ In our case, the MHP powders were synthesized and kept under an inert atmosphere, but the NMR rotor was packed in air, which could induce degradation processes even for the 50% and 75% FA^+^ powder mixtures. Furthermore, the fact that we do not employ solvent-assisted milling can influence the stability of the final powders under ambient conditions.

**Figure 3:**
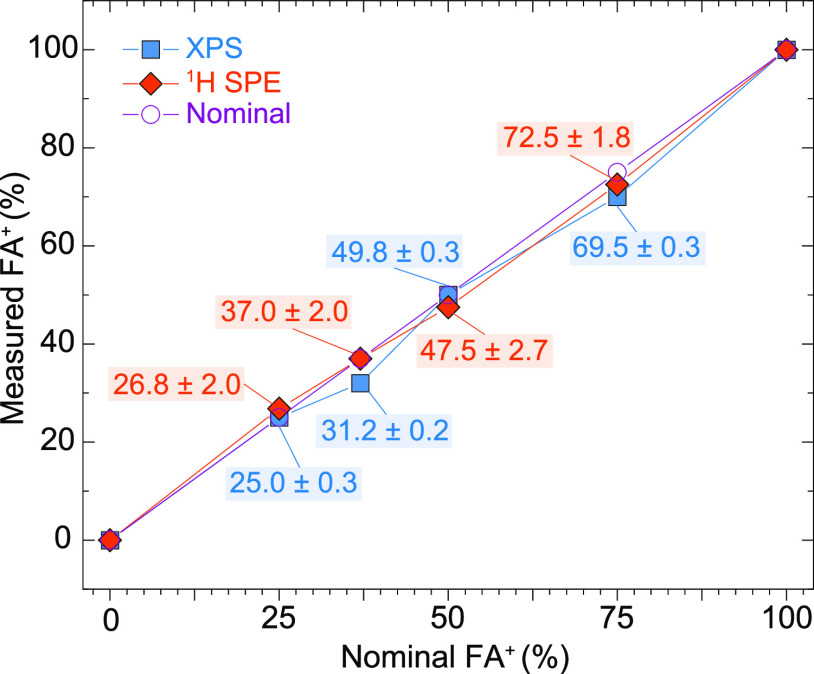
Plot comparing the measured FA^+^ (%) obtained from XPS and ^1^H SPE NMR spectra as a function of the nominal FA^+^ (%) as added during mechanochemical synthesis.

For the MA^+^:FA^+^ ratio quantification, signals were fitted using DMFit^59^ and ssNAKE.^58^Table S2 compares the results obtained from the direct excitation ^13^C MAS NMR and the ^1^H MAS NMR spectra. The MA^+^:FA^+^ ratios obtained from ^1^H one pulse NMR are considered the most accurate quantification method in this study. This choice is based on the more symmetric and, therefore, more reliable line shapes compared to ^13^C NMR, as evidenced by the close alignment of the results with the nominal powder stoichiometries. For a nominal FA^+^ (%) = 25, 37, 50, and 75, the measured ^1^H NMR FA^+^ (%) are 26.8 ± 2.0, 37.0 ± 2.0, 47.5 ± 2.7, and 72.5 ± 1.8, respectively (see Figure 3).

In order to compare the cation ratios obtained from the bulk of the samples using MAS ssNMR, X-ray photoelectron spectroscopy (XPS) was employed, particularly for the N 1s and C 1s core levels of the FA^+^ and MA^+^ cations. XPS enables the observation of chemical shift effects, with the potential for up to 10 eV shift in the peak position of a specific core level of an element.^72^ This shift reflects variations in their local chemical environment, a phenomenon particularly evident in the case of MA^+^ and FA^+^ molecules.^72^ For better manipulation of the perovskite powders, XPS measurements were conducted on the pressed powders (also referred to as pellets and presented in Figure 1h). Figure 2b shows the N 1s core-level spectra of the four MCS MA_1–*x*_FA_*x*_PbI_3_ (*x*: 0.25–0.75) pressed powders, with two distinct chemical species detected. Peaks at 400.2 ± 0.1 eV are assigned to FA^+^ (**N**H_2_-C) cations, while peaks at 401.8 ± 0.1 eV are attributed to MA^+^ (**N**H_3_-C) cations.

Similarly, Figure S2 displays the C 1s core-level spectra revealing three chemical species: C–C–H at 284.85 eV (adventitious carbon), carbon bonded to nitrogen (**C**-NH_2_) in FA^+^ at 288.1 ± 0.1 eV, and (**C**-NH_3_) in MA^+^ at 286.2 ± 0.1 eV. Due to the influence of the carbon signal from the environment, the C 1s core levels are not utilized for cation ratio analysis.^73^ Nonetheless, the significant changes in the peak intensity of the N 1s core levels can be employed to estimate the cation ratio MA^+^:FA^+^. Note that, in solution-based processes where small amounts of MA^+^ are typically used, it becomes challenging to identify MA^+^ in a spectrum dominated by FA^+^ content.^46^ The latter is because the FA^+^ (NH_2_)_2_–CH^+^ cation presents two contributions equivalent to N–C bonding, resulting in a higher peak intensity compared to MA^+^ (CH_3_–NH_3_)^+^, which has a single N–C bond (Table S4). Complementary information regarding XPS survey spectra, the fitting, and analysis of all samples can be found in Figures S3–S10 and Table S5.

The evolution of the MHP-pressed powders’ cation ratio determined by XPS is depicted in Figure 3, showing its comparison with ^1^H MAS NMR and the expected cation ratio based on the nominal values. The results from ^1^H MAS NMR align well with the nominal ratios and are consistent with previous studies on MA_1–*x*_FA_*x*_PbI_3_ perovskites utilizing a mechanosynthesis process with planetary ball-milling and cyclohexane as a milling agent.^16^ However, the values obtained from XPS show slightly lower FA^+^ content for the nominal 37 and 75%, giving 31.5 and 69.5% FA^+^ content, respectively. The slight deviations from the nominal ratio could be attributed to measurement errors, accurate selection of peak fitting areas and models, or incomplete solid-state reactions in our ball-milled approach (Figures S3–S10 and Table S5).^74^ Additionally, undesired decomposition of perovskites triggered by X-ray exposure under ultrahigh vacuum conditions could affect quantification, potentially leading to deviations in results compared to ^1^H MAS NMR.^75^

In this case, the close correlation between ^1^H MAS NMR, XPS, and the nominal values for the MCS MA_1–*x*_FA_X_PbI_3_, suggests that one can use XPS to determine the cation ratio also on powders, however, with caution for possible differences between surface and bulk in the case of large powder clusters. ^13^C direct excitation in ssNMR turned out to be unsuitable for quantitative analysis but could still identify secondary phases as explained earlier (see Figure S1). Based on this analysis and the Pb/I ratio obtained from the XPS analysis (varying between 2.9 and 3.1), we can estimate the actual composition of the mechanosynthesized powders as ∼MA_0.73_FA_0.27_PbI_3_, MA_0.63_FA_0.37_PbI_3_, MA_0.53_FA_0.47_PbI_3_, and MA_0.27_FA_0.73_PbI_3_.

### III.III Structural and Optical Characterization of MCS MA_1–*x*_FA*_x_*PbI_3_

**Figure 4:**
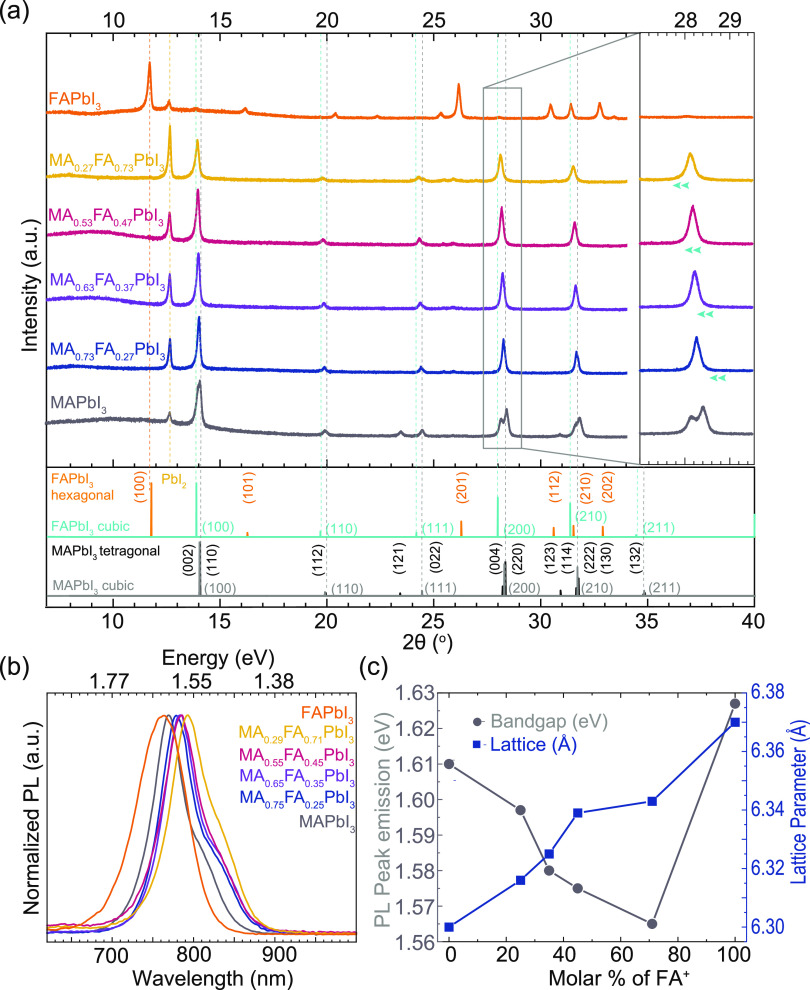
(a) X-ray diffraction (XRD) patterns of MCS MA_1–*x*_FA_*x*_PbI_3_ (*x*: 0–1) pressed powders along with the reference patterns of expected crystalline phases. A zoomed-in region demonstrates the shift toward lower angles as the FA^+^ content increases. (b) Normalized PL spectra of MA_1–*x*_FA_*x*_PbI_3_ (*x*: 0–1) pressed powders. (c) Estimation of the bandgap (E_g_) based on the PL peak emission, revealing a red shift with increasing FA^+^ molar% up to 70%. This red shift also corresponds to the expansion of the lattice parameters with increasing the FA^+^ molar content (see Table S6). Note: The lines in (c) are only provided for guidance purposes.

Figure 4a displays the structural characterization of the MCS MA_1–*x*_FA_*x*_PbI_3_ powders as well as the single-cation MCS MAPbI_3_ and FAPbI_3_ powders. Here, all of the cation ratios are corrected based on the analysis in the previous section. The XRD plot of the MCS MAPbI_3_ exhibits a distinctive peak at 23.4 2θ° (121), which is indicative of the tetragonal phase. Furthermore, a clear peak splitting at around 28.4 2θ° (004) and (220) confirms the presence of the tetragonal phase.^76^ It has been reported that both tetragonal and cubic phases of MAPbI_3_ can coexist at room temperature despite the transition from the β-phase to the α-phase occurring at approximately 57 °C.^77,78^ To investigate this transition, we conducted in situ temperature-dependent XRD (Figure S11).

Above 65 °C, the XRD plot shows a single peak at around 28.3 2θ°, corresponding to the cubic phase, whereas a reversible transition or double peak splitting is observed upon cooling down to 30 °C. This observation provides insights into the predominance of the tetragonal photoactive β-phase at room temperature in the bulk MAPbI_3_ MCS powder, as well as of the purity of the powders, showing the phase transition at the expected reported temperature for powder samples.^78^ The SEM cross section of the pressed MCS MAPbI_3_ powders is presented in Figure 1e–g.

The case of FAPbI_3_ is a bit more intriguing because the hexagonal δ-phase to the cubic α-phase transition occurs at temperatures above 150 °C, as shown in Figure S12. This transition can also be induced by pressure.^79,80^ Immediately after mechanochemical synthesis, FAPbI_3_ presents the δ-phase (yellow phase).^11^ However, during the powder pressing, a constant pressure of approximately 158 MPa is applied likely inducing the phase transformation of the photoactive phase of FAPbI_3_, as observed in the small signals in the XRD plot of the (100) family planes, indicating the coexistence of the hexagonal and cubic phases at room temperature (Figure 4a).^78^ Correspondingly, a noticeable change in color from yellow to brownish-pressed powder was observed (Figure S12). Similar to MAPbI_3_, the phase transition of FAPbI_3_ is reversible. However, in the case of the pressed powder, the transition did not occur immediately, suggesting improved stability of the FAPbI_3_ pressed powder to maintain its cubic α-phase compared to thin films.^81^

To further examine the stability of the cubic phase in mixed-cation perovskites, we performed the in situ annealing during XRD from room temperature up to 180 °C on the pressed powder with an MA^+^:FA^+^ molar ratio of 47/53. Figure S13 demonstrates that there is no change or shift observed in the (100) and (200) planes at different temperatures, indicating high stability of the cubic phase of mixed-cation perovskite even at temperatures as high as 180 °C. Any observed shifts in the XRD pattern may be attributed to changes in alignment due to the measurement conditions.

The expected bandgaps for the α-cubic and β-tetragonal phases of MAPbI_3_ are approximately 1.67 and 1.60 eV, respectively. In contrast, for FAPbI_3_, the α-cubic phase is expected to be close to 1.48 eV.^81,82^ Therefore, mixed-cation perovskites MA_*x*_FA_1–*x*_PbI_3_ are assumed to exhibit red-shifted bandgaps as the amount of FA^+^ increases.^7^ Here, we employed steady-state photoluminescence (PL) with a 520 nm laser to analyze the emission of the MHP-pressed powders (Figure S14). For a better comparison, the normalized PL emission is presented in Figure 4b. A shoulder on the red side of the main PL emission spectra is noticeable, which could be attributed to reabsorption due to the high thickness of the bulk pellet.^83^

Figure 4c compares the bandgap (*E*_g_) values obtained from the PL peak emission of each pellet with the lattice parameters estimated from the XRD plots (Tables S6 and S7). For instance, the E_g_ values for MAPbI_3_, MA_0.73_FA_0.27_PbI_3_, MA_0.63_FA_0.37_PbI_3_, MA_0.53_FA_0.47_PbI_3_, and MA_0.27_FA_0.73_PbI_3_ are 1.61, 1.60, 1.58, 1.57, and 1.56 eV, respectively. The BG and red shift with increasing FA content are consistent with values reported in the literature.^7^ Still, the case of FAPbI_3_ is distinctive due to the coexistence of black and yellow phases in the pressed powders, resulting in a bandgap of 1.63 eV. Similarly, the successful incorporation of the larger FA^+^ cation into the lattice expands the volume of the unit cell, which is reflected in the increased lattice parameter (Figure 4c).

### III.IV From MCS Pressed Powders to Thin Films

**Figure 5:**
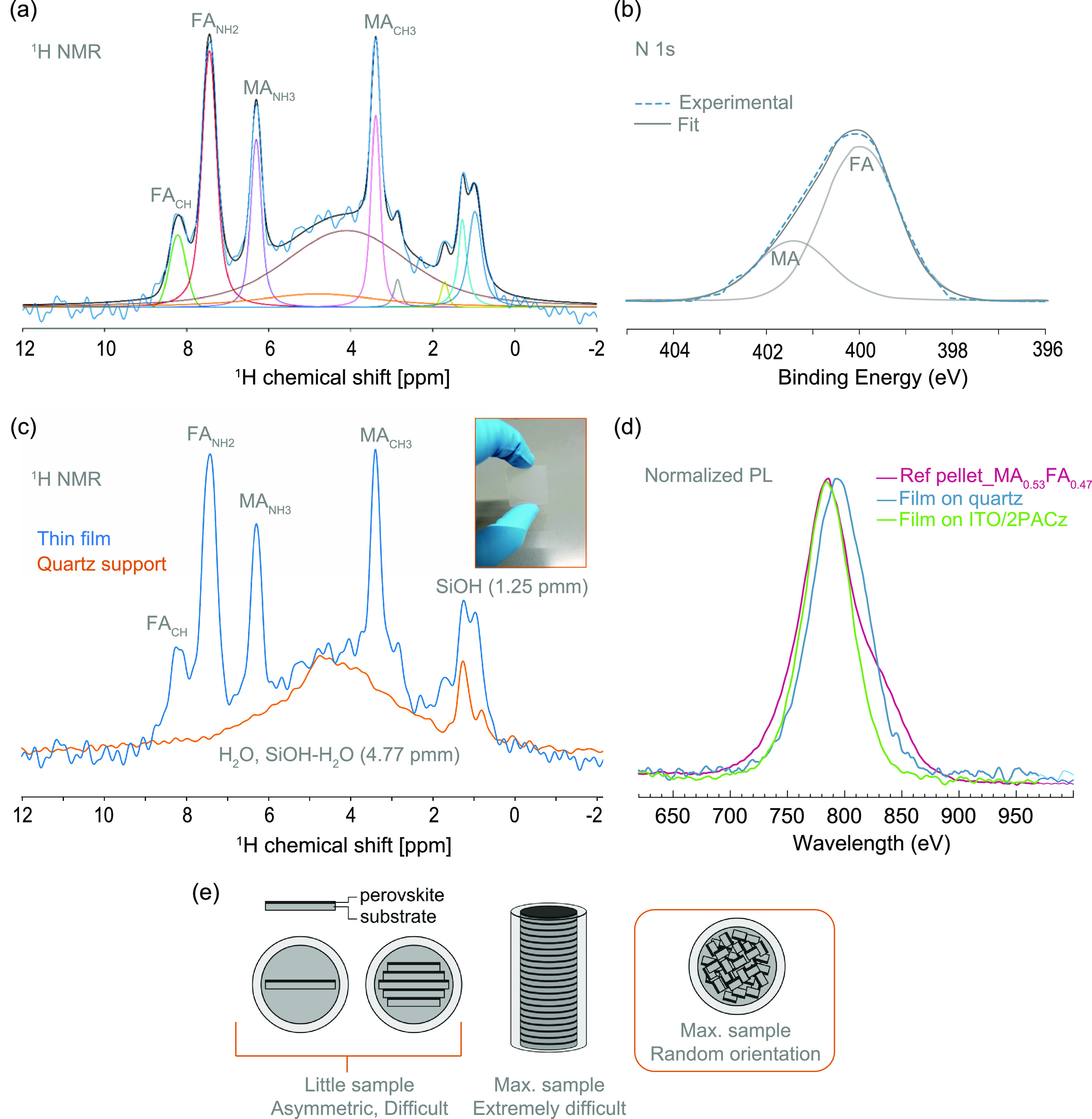
(a) ^1^H MAS NMR spectrum obtained from a single MA_1–*x*_FA_*x*_PbI_3_ thin film, ∼800 nm thick, grown on a 0.2 mm thick quartz substrate employing PLD. Quantitative analysis from the spectrum indicates an MA^+^:FA^+^ ratio of 0.39:0.61 for the PLD-growth film; (b) XPS spectra of N 1s core levels corresponding to an MA_1–*x*_FA_*x*_PbI_3_ thin film grown under the same conditions. Quantitative analysis from XPS indicates an MA^+^:FA^+^ ratio of 0.44:0.56 in the PLD-growth film. (c) ^1^H MAS NMR of the quartz substrate spectrum (1 × 1 in.^2^, 0.2 mm thick, crushed using a mortar and pestle in air) compared to the thin film. (d) Normalized photoluminescence spectra of the reference pellet of MA^+^:FA^+^ 53:47 in comparison to two thin films grown under the same PLD conditions, one on quartz substrate (blue) and one on contact layers ITO/2PACz (green). (e) Several options to introduce thin films into an NMR rotor, highlighting the chosen option, using a maximum filling of the rotor with samples in a random orientation. The other options are deemed to be difficult to pack the rotor well enough for it to spin properly.

Employing previously optimized thin film growth conditions^35^ and using scanning mode to homogeneously cover a 1 × 1 in.^2^ substrate, MA_1–*x*_FA_*x*_PbI_3_ thin films were grown by PLD from the MCS pressed powders. In this case, the pellets with excess organics from Figure 1d are employed. Contrary to our previous study^35^ where multiple samples were scraped off to fill the rotor with the powder from the thin films, here we test ^1^H MAS NMR of a single ∼800 nm-thick MA_1–*x*_FA_*x*_PbI_3_ film coated on a very thin 0.2 mm quartz substrate, to keep the relative amount of perovskite in the sample as high as possible. This substrate thickness facilitates crushing the sample into powder particles using a mortar and pestle, to optimally fill the NMR rotor (see Figure 5d) and ensure smooth spinning. Figure 5a,b displays the ^1^H MAS NMR and XPS N 1s core-level spectra of the MA_1–*x*_FA_*x*_PbI_3_ thin film and its deconvolution.

A PLD target containing 25% FA^+^ (75% MA^+^) is employed as the single source. This FA^+^ % is chosen to obtain thin films with ∼50–60% FA^+^ when employing the scanning PLD mode.^35^ Quantitative analysis from the ^1^H MAS NMR spectrum indicates an MA^+^:FA^+^ ratio of 0.39:0.61 while quantitative analysis from XPS indicates an MA^+^: FA^+^ ratio of 0.44:0.56 in the PLD-growth film. The cation ratios from XPS and NMR for the films differ a bit more than for the case of the MCS powders alone (Figure 5). This is not surprising for two main reasons: 1. the possible difference in the thin film surface composition compared to the bulk and 2. the additional background signal in the ss-NMR spectrum (Figure 5c) largely related to the quartz support. This background makes up 61.8% of the total integrated signal, compromising the fitting of the spectrum compared to the ^1^H spectra of the pressed powders, where the signals from the MA^+^: FA^+^ cations were well resolved (Figure 2a). 3. The influence of substrate-dependent growth of vapor-deposited perovskites, which, in turn, affects the sticking and incorporation rate of organic molecules resulting in variable organic cation ratios.^84,85^ A thin film grown on ITO/2PACz would be expected to exhibit a cation ratio closer to 50:50 (Figure 5d) with the corresponding PL emission slightly blue-shifted with respect to the thin film grown on bare quartz substrates with 39:61 MA^+^: FA^+^ ratio.

The ^1^H spectrum of the thin film exhibits extra signals in the region between 0 and 5 ppm, overlaid with the cation peaks in that region. Signals in the region between 2 and 5 ppm are attributed to hydroxyl groups in the quartz substrate^84,85^ (see Figure 5c). Figure S15 shows a spectrum of two different SiO_2_ compounds (quartz sand and silica gel) taken at identical experimental conditions for comparison. Although these compounds are hygroscopic, we expect any absorbed water molecules to be rather mobile. In this case, associated peaks would be narrow enough to stand out in the spectrum. We therefore believe it is unlikely there is a significant amount of water present although the presence of less mobile water molecules cannot be completely ruled out. The origin of the signals arising between 0 and 1 ppm is so far uncertain. To the best of our knowledge, these signals do not correspond to any MHP, MHP precursor, or remaining solvents used to clean the rotor (IPA, EtOH) before packing the sample (note that the thin film does not undergo any solution-process step either).^16,86^ If these signals are the consequence of organic byproducts of the production method or degradation of the proper organic cations, this would also influence the estimation of the cation ratio MA^+^: FA^+^ of the thin film. Attempts were made to remove the unwanted signals using either a Hahn Echo or the EASY pulse sequence (see Figure S16).^87,88^ However, both affected the perovskite signals as well, with different effects on the various MA^+^ and FA^+^ peaks, and thus, it was decided not to use them. Despite the presence of the extra signals, the MA^+^:FA^+^ ratio estimated from deconvoluting the ssNMR spectrum is 39:61, which corresponds to the expected stoichiometry of the films when employing the PLD scanning mode for thin film growth on bare silica.^35^

Considering the aim to establish a characterization protocol in this work, it is worthwhile to consider the variables influencing the quality of the NMR spectrum in Figure 5. First, it should be noted that it took only 2 h to acquire the spectrum of both the sample and the empty rotor. Given that the spectrum has a signal-to-noise (S/N) of 50, this means this technique allows for a decent throughput. For general application, it is worthwhile to consider the effect of the magnetic field strength *B*_0_, where S/N scales with *B*_0_^7/4^, and that of the absorption layer-to-support ratio *r*, where S/N scales with *r*/(*r* + 1) due to the support occupying part of the measurable volume. In practical terms, this means that similar spectra would, for example, take 28 h for the same ratio at a field of 400 MHz or 8 h for half the ratio at the same field (see also Table S3). Depending on the setup, which has no other particular requirements, the signal-to-noise can realistically be doubled or even quadrupled although it should be noted that convolution of nonperovskite signals, not S/N, is the current bottleneck in accurate quantification. The sensitivity of ^1^H NMR also allows for the tracking of thin film degradation on a time scale of hours although in this work only slight degradation was observed after a month (see Figure S17). Given the gyromagnetic ratio and natural abundance of ^13^C, direct excitation spectra of that isotope take of the order of 10 million times as long as ^1^H spectra under identical conditions. As this means even an S/N of 10 would take 10 years using a similar field, it is safe to conclude that this is unfeasible for any realistic application even without considering the longer *T*_1_’s (Table S1). Cross-polarization (CPMAS) would increase the sensitivity of ^13^C, but the quantitative version of this pulse sequence requires significant time and effort to set up and validate.^87^ DNP is even harder to do quantitatively and would, due to the short *T*_1_’s compared to the rate of spin diffusion in perovskites, only achieve limited enhancement (see, e.g., Hanrahan et al.^37^). These techniques are therefore not well-suited for a standardized protocol.

**Figure 6:**
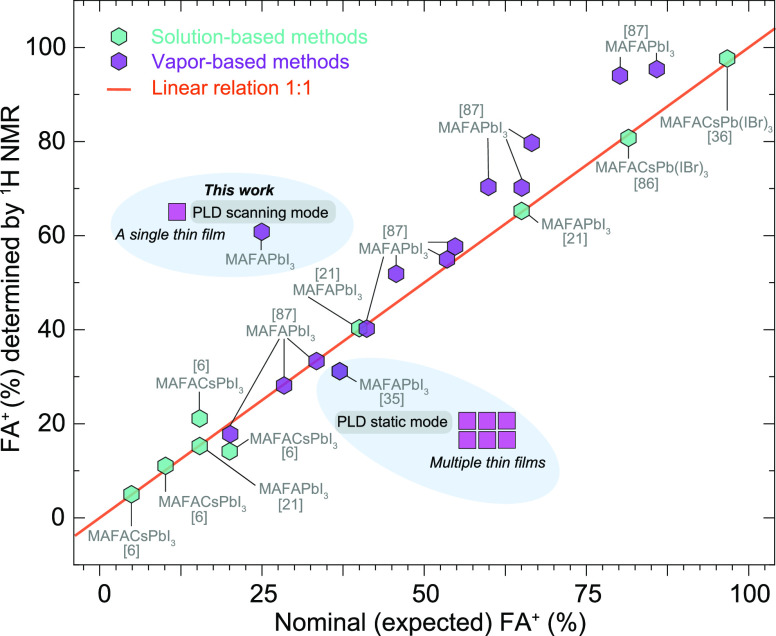
Comparison between thin film cation ratio quantification using a single thin film (this study) and multiple thin films (previous study) by PLD with other solution and vapor deposition methods reporting the estimation of cation ratios using ^1^H NMR. Note: for the *x*-axes, nominal values refer to FA% used in solution processes, while expected values refer to source composition or QCM calibration values for vapor-deposited perovskites.

Using these results and previously reported organic cation ratios in MHP films prepared by different methods and measured by NMR, a comparison between precursor ratio vs film composition is presented in Figure 6 for MHP compositions containing FA^+^ and MA^+^ cations. The data are categorized into solution-processed vs vapor-processed MHP. As observed in Figure 6, nominal precursor ratios in the solution process result in films with virtually equal organic composition (or ratio).^36,44^ This linear relation varies for compositions containing triple cations when the incorporation of Cs is >5%.^6^ However, for vacuum-based processes, the estimation deviates from the 1:1 line when the nominal FA^+^ content is >55% for coevaporation due to more difficult incorporation and sticking of MAI, for FA^+^ < 50% the stoichiometry is maintained.^89^ For the case of static PLD (i.e., no substrate scanning and just directional deposition), near stoichiometric transfer from the source to film is achieved,^35^ but for large area depositions, for example, by scanning the substrate (scanning PLD), a distinctive source to film organic ratio is determined. This is likely an effect of cation distribution in the dynamic plasma plume, sticking of the molecules (as mentioned before for the case of MA^+^ and FA^+^) and different deposition rates due to scanning on a large area coating, all together modifying the growth mode and final thin film composition.^35^ Note that for PVD methods, the perovskite composition and substrate type considerably influence the cation ratio of the final thin film due to variations in substrate polarity and sticking of molecules.^90,91^ Hence, this investigation underscores the significance of accurately calibrating and quantifying the organic cation ratio both in the PVD target and in thin films, which varies depending on the deposition conditions and substrate type.

### III.V Pros and Cons of Single-Thin Film Measurements via ss-NMR

#### III.V.I Pros

- Nondestructive analysis: Analyzing thin films without removing them from the substrate (without scratching them off) allows for the observation of strain effects and phase stabilization induced by the substrate or contact type. This method also enables the study of interface engineering and passivation strategies, such as the use of buffer templates that influence the growth orientation and morphology of MHP films.
- Feasibility with thin substrates: We demonstrate the feasibility of measuring 800 nm-thick films on a 0.2 mm thick quartz substrate in less than 2 h. For thinner films (<800 nm) on device stack substrates, glass substrates as thin as 0.2 mm, coated with an ∼100 nm layer of ITO along with the corresponding contact material (HTL or ETL) should be used.
- Extended to systems with inorganic cations: The method is not restricted to organic cations (those with hydrogen) but can be applied to systems where inorganic cations (those without hydrogen) are also present. For this, one can compare the measurements to a reference compound with a known amount of protons, providing a benchmark for quantification.

#### III.V.II Cons

- Cost: Thin substrates (0.2 mm) are more costly and difficult to handle as compared to traditional 0.5 to 1.1 mm glass/ITO substrates.
- Signal interference: Extra signal from the substrate (or contact layers, such as self-assemble monolayers, SAMs^92^) can interfere with measurements even when attempts were made to remove the unwanted signals using either a Hahn Echo or the EASY pulse sequence.
- Acquisition time: Obtaining ^13^C spectra with sufficient signal-to-noise ratio for quantification purposes requires significant acquisition time and effort to set up and validate.

## IV Conclusions

Solid-state NMR was used to quantify the organic cation MA^+^:FA^+^ ratio of bulk MA_1–*x*_FA_*x*_PbI_3_ perovskite (pressed) powders and thin films. For this, ^1^H NMR experiments were sensitive enough to record quantitative spectra of a single crushed thin film within a couple of hours. The actual compositions of the MCS (pressed) powders were found to be close to their nominal stoichiometries. In contrast, the thin film showed a slight deviation from the expected values as well as partially unexplained extra signals. This discrepancy is likely attributed to the significant contribution of the quartz substrate to the spectrum, owing to the substantial ratio between the substrate and perovskite coating. Although ^13^C spectra could be recorded for the pressed powders, their sensitivity is insufficient for accurate cation ratio determination from a single crushed thin film in any reasonable time frame. As a result, ^13^C experiments are not recommended as a standard procedure. Characterizing mechanochemically synthesized perovskites with XPS ensures the homogeneous and reliable composition necessary for reproducible thin film growth via single-source vapor deposition methods, enabling accurate tuning of thin film composition and providing cation ratio estimates when ssNMR is not readily accessible. Despite being a surface-sensitive technique, XPS results can, in cases such as powders, closely reflect bulk composition. However, we recommend calibrating XPS values using techniques such as ssNMR. Finally, the material properties of the bulk MHP correspond to those reported in the literature, indicating that mechanochemical synthesis of MHP is a promising and alternative option for preparing these reference perovskite powder materials. The resulting high-quality perovskite powders can function as precursors for thin film formation, such as targets for PVD methods. We unveiled the actual cation ratio in the presynthesized perovskite material, establishing a foundation for subsequent correlation with the cation ratio of, for example, PVD targets and the grown perovskite films under distinctive PVD deposition conditions.

## Supporting Information Available

Additional information from NMR experiments including ^13^C MAS NMR, ^1^H MAS NMR spectra of different quartz supports, extra ^1^H MAS NMR spectra of the thin film measured at different pulsed sequences and time, *T*_1_ estimated values, and estimated times for a S/N of 100; detailed description of XRD temperature dependence analysis, lattice constant calculation, and additional XPS analysis (PDF)
