# Supplementary material for: Quantifying Organic Cation Ratios in Metal Halide Perovskites: Insights from X-ray Photoelectron Spectroscopy and Nuclear Magnetic Resonance Spectroscopy
Source: Chem Mater. 2024 Jul 5;36(14):6912–24. doi: 10.1021/acs.chemmater.4c00935 (PMC11270747; doi:10.1021/acs.chemmater.4c00935)
Supplement: Supplementary file 1 — cm4c00935_si_001.pdf [file cm4c00935_si_001.pdf]

Supporting Information

Quantifying Organic Cation Ratios in Metal Halide Perovskites: Insights from XPS and NMR Spectroscopy

Tatiana Soto-Montero,[†] *Suzana Kralj,* [†] *Jennifer S. Gómez,* [⊥] *Jop W. Wolffs,* [⊥] *Nathan Rodkey,* [†, X] *Arno P. M. Kentgens*[*, ⊥]*, Monica Morales-Masis*[*, †]

[†]MESA+ Institute for Nanotechnology, University of Twente, Enschede 7500 AE, The Netherlands.

[⊥]Institute for Molecules and Materials, Radboud University, 6525 AJ Nijmegen, The Netherlands

[X]Instituto de Ciencia Molecular, Universidad de Valencia, 46980 Paterna, Spain

Figure S1*: ^{13}C MAS NMR spectra of mixed MHP.*
Figure S2*: C 1s core levels spectra of mixed MHP.*
Figure S3*: Survey spectral region of the MHP pellets.*
Figures S4-10*: Fitting of the high resolution I 3d, Pd 4f, N1s, and C1s core levels spectrum of $MAPbI_3$, $FAPbI_3$, $MA_{0.75}FA_{0.25}PbI_3$, $MA_{0.63}FA_{0.37}PbI_3$, $MA_{0.50}FA_{0.50}PbI_3$, $MA_{0.25}FA_{0.75}PbI_3$, MAI and FAI, respectively.*
Figures S11-13*: Temperature dependence XRD analysis of the $MAPbI_3$, $FAPbI_3$, and $MA_{0.55}FA_{0.45}PbI_3$ pellet, respectively.*
Figure S14*: Photoluminescence set up to measure the MHP pellets.*
Figure S15*: ^{1}H MAS NMR spectra of quartz sand, quartz silica gel, and quartz support.*
Figure S16*: ^{1}H MAS NMR spectra of the $MA_{45}FA_{55}PbI_3$ thin film with substrate, showing the effect different pulse sequences meant to suppress unwanted signals.*
Figure S17*: ^{1}H MAS NMR DEPTH spectra of the $MA_{45}FA_{55}PbI_3$ thin film with substrate measured at separate dates, demonstrating minor sample instability.*
Table S1: *Longest T_1values determined in the MAS NMR experiments of the four mixed $MA_{1-x}FA_xPbI_3$ samples and the thin film.*
Table S2*: Quantification of FA/MA ratios using the direct ^{13}C MAS NMR and the ^{1}H MAS NMR spectra.*
Table S3: *Estimated time required to achieve an S/N of 100 in a ^{1}H MAS spectrum.*
Table S4*: N atomic percentage by XPS-based and experimental cation ratio of stoichiometric pellets.*
Table S5*: Spectra features table of Pb 4f, I 3d, and C 1s core levels.*
Table S6*: Estimation of the lattice parameters using X-ray diffraction data.*
Table S7*: Bandgap of thin pellets from photoluminescence measurements.*
Tables S8-14*: Stoichiometric Halide Perovskites Pellets additional information.*
Table S.15*: Pellets density estimation after 30min at 5 metric tons (~158.66 MPa)*

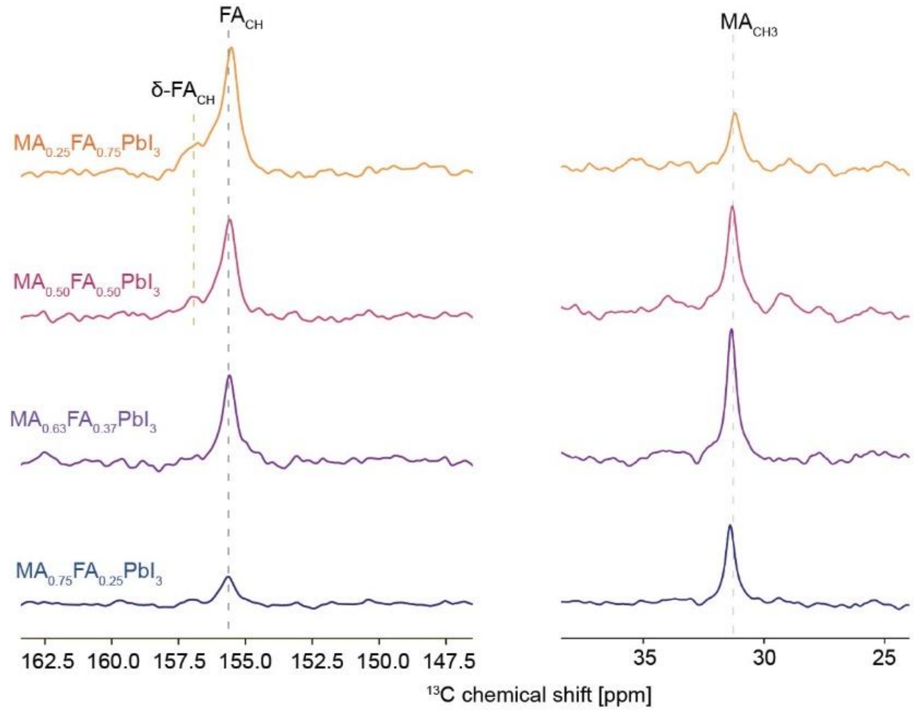

Figure S1: ^{13}C CPMAS NMR spectra of mixed MHP showing the resonances for the **C**H group of the FA^+ cation at 155.5 ppm and the **C**H_3 group of the MA^+ cation at 31.3 ppm. The yellow dashed line indicates a shoulder that could be attributable to either natural line shape asymmetry or the presence of the yellow δ-phase.

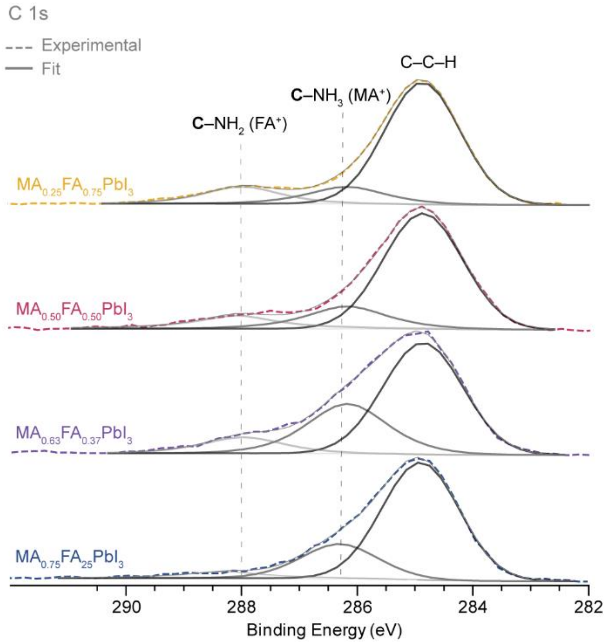

Figure S2: C 1s core levels displaying signal contributions from the organic cations and adventitious carbon.

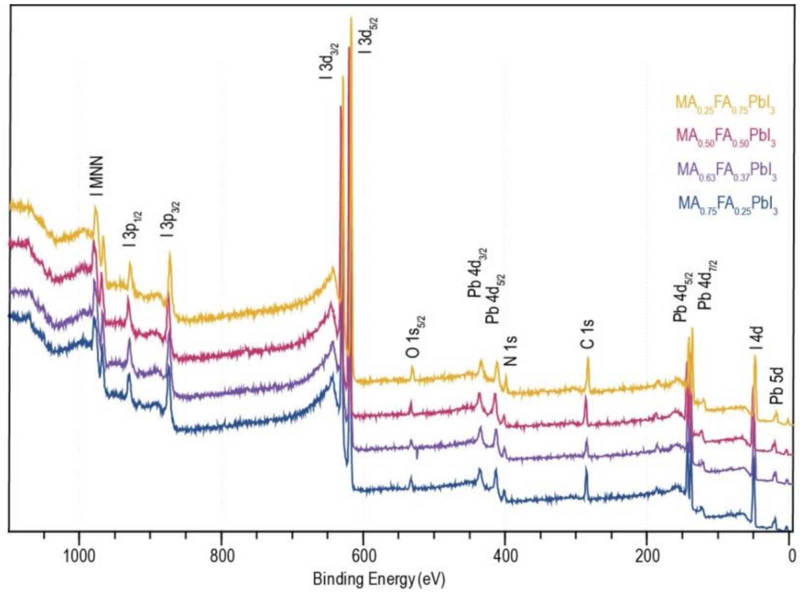

Figure S3: Survey spectral region displaying major elements (Pb, I, C, and N) and Pb 4f, I 3d, C 1s, N 1s, Pb 4d, and Pb 5d core levels of the MHP pellets.

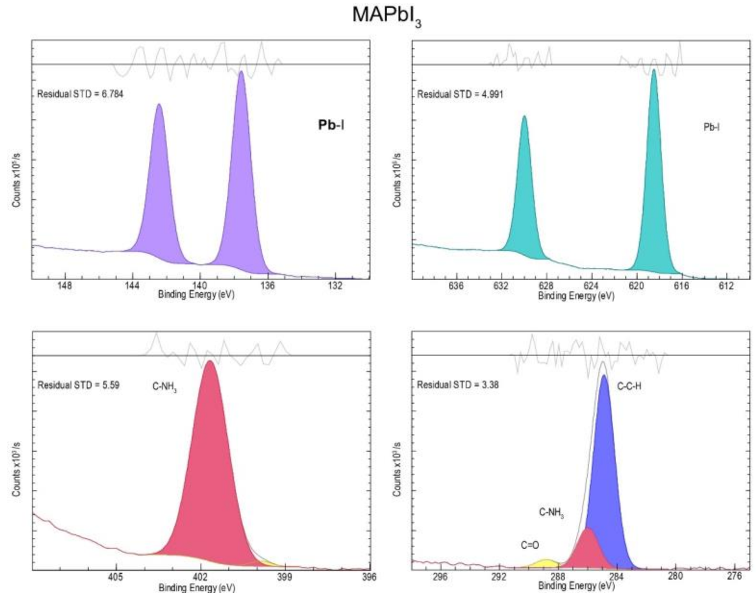

Figure S4: Fitting of the high resolution I 3d, Pd 4f, N1s, and C1s core levels spectrum of $MAPbI_3$.

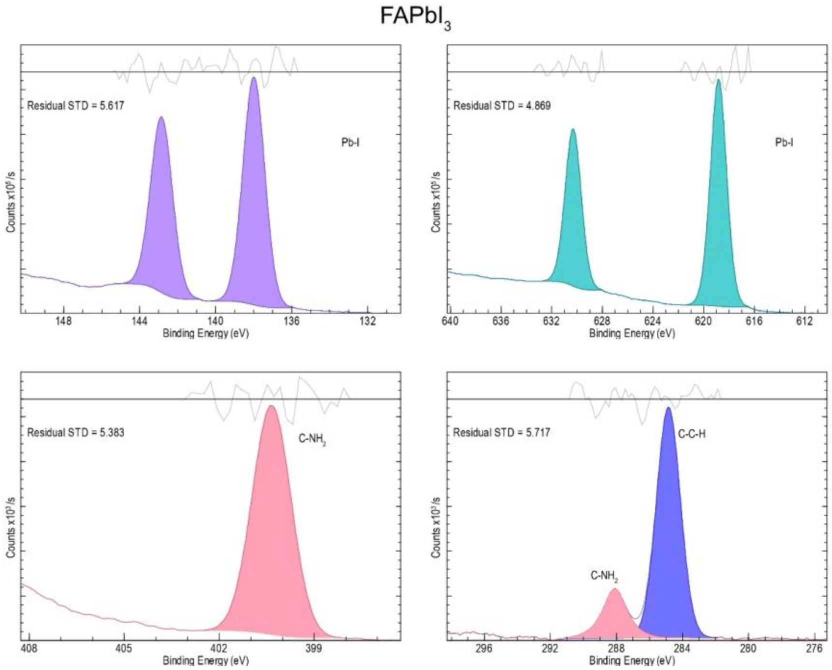

Figure S5: Fitting of the high resolution I 3d, Pd 4f, N1s, and C1s core levels spectrum of $FAPbI_3$.

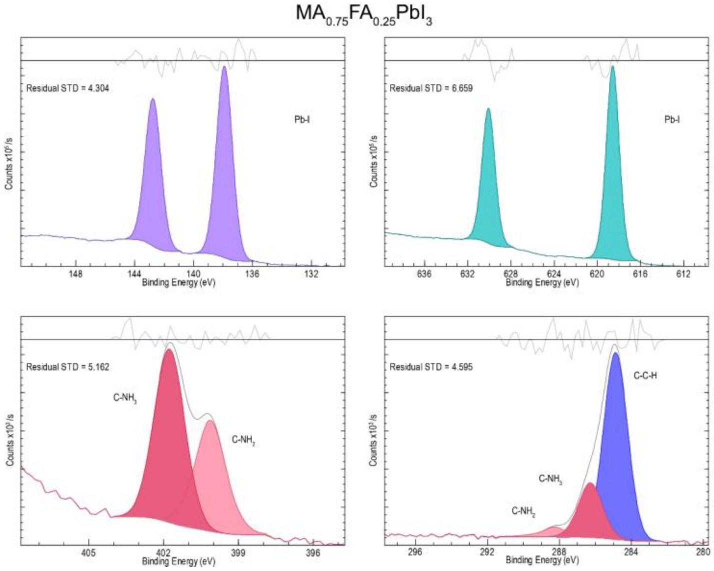

Figure S6: Fitting of the high resolution I 3d, Pd 4f, N1s, and C1s core levels spectrum of $MA_{0.75}FA_{0.25}PbI_3$.

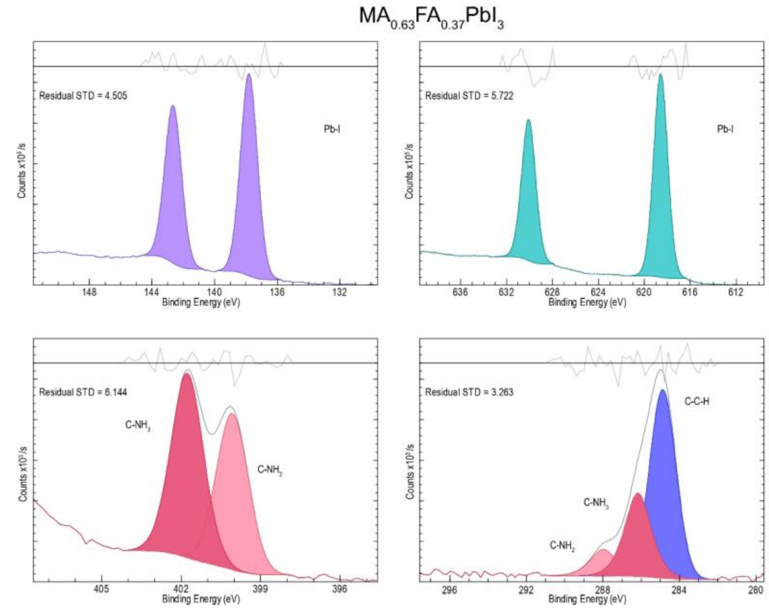

Figure S7: Fitting of the high resolution I 3d, Pd 4f, N1s, and C1s core levels spectrum of $MA_{0.63}FA_{0.37}PbI_3$.

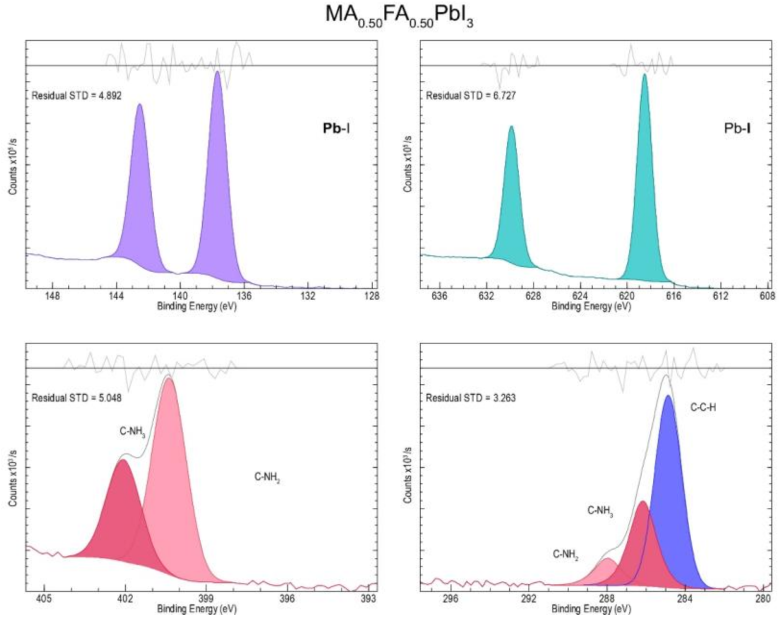

Figure S8: Fitting of the high resolution I 3d, Pd 4f, N1s, and C1s core levels spectrum of $MA_{0.50}FA_{0.50}PbI_3$.

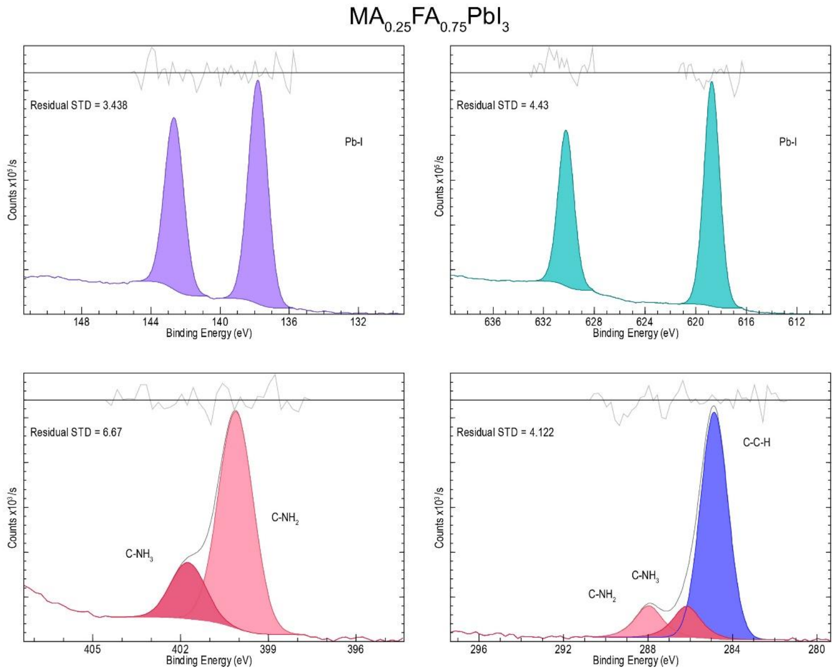

Figure S9: Fitting of the high resolution I 3d, Pd 4f, N1s, and C1s core levels spectrum of $MA_{0.25}FA_{0.75}PbI_3$.

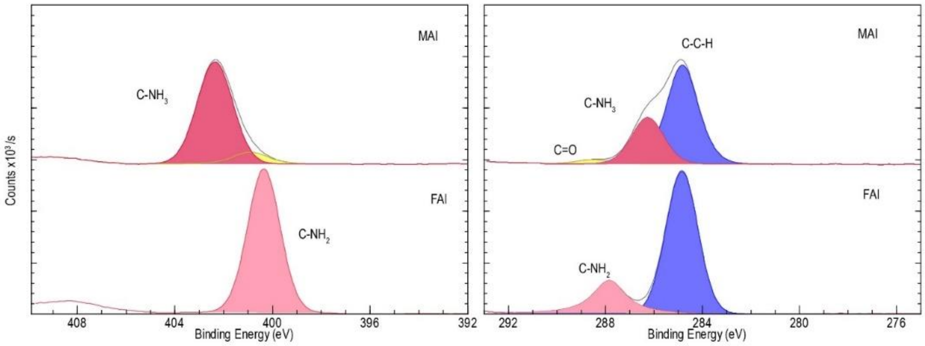

Figure S10: Fitting of the high resolution N1s, and C1s core levels spectrum of the precursors MAI and FAI (>99.99% Greatcellsolar).

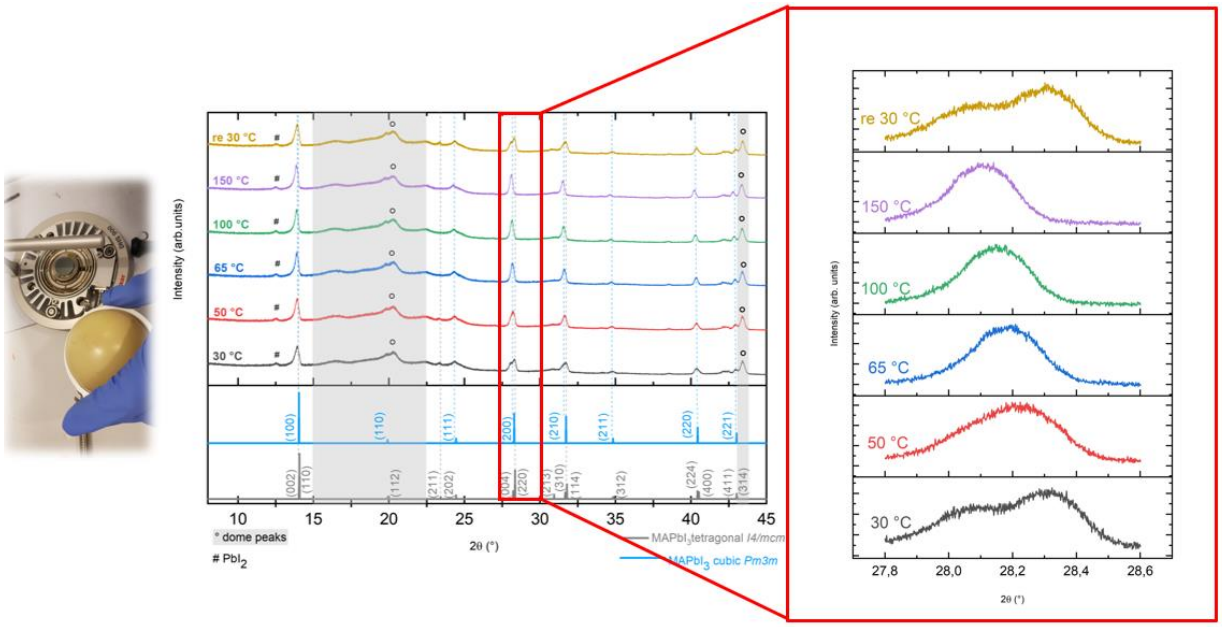

Figure S11: Temperature dependence XRD analysis of the $MAPbI_3$ pellet.

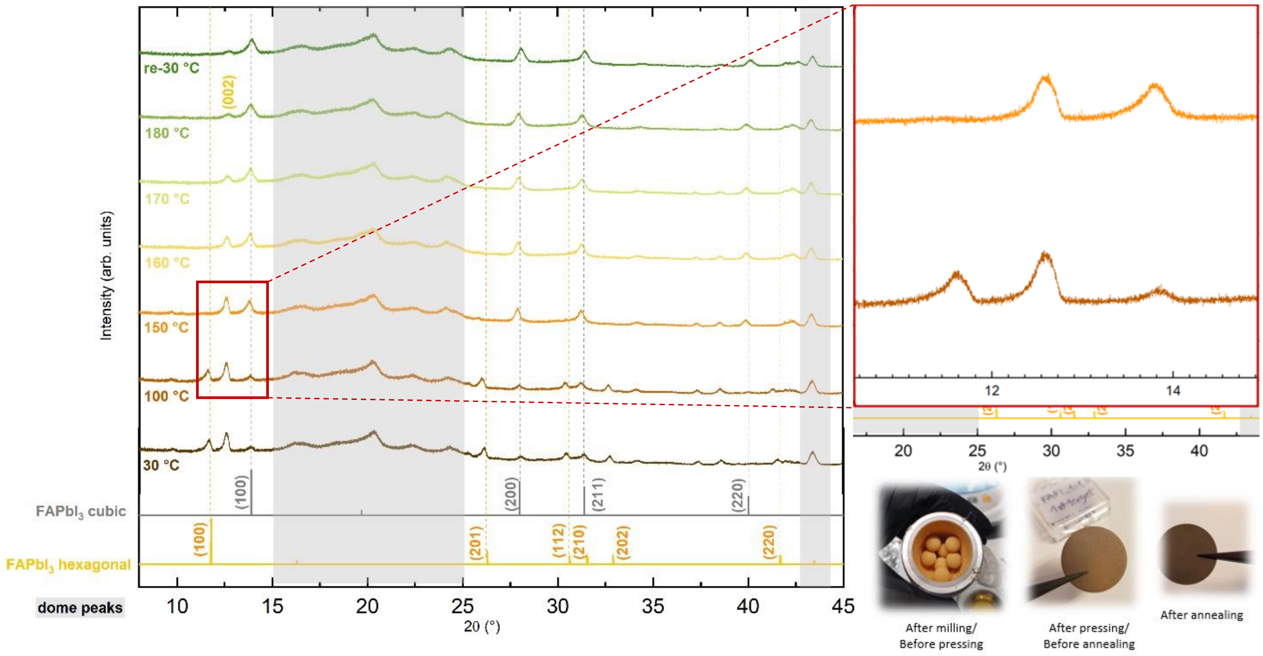

Figure S12: Temperature dependence XRD analysis of the $FAPbI_3$ pellet.

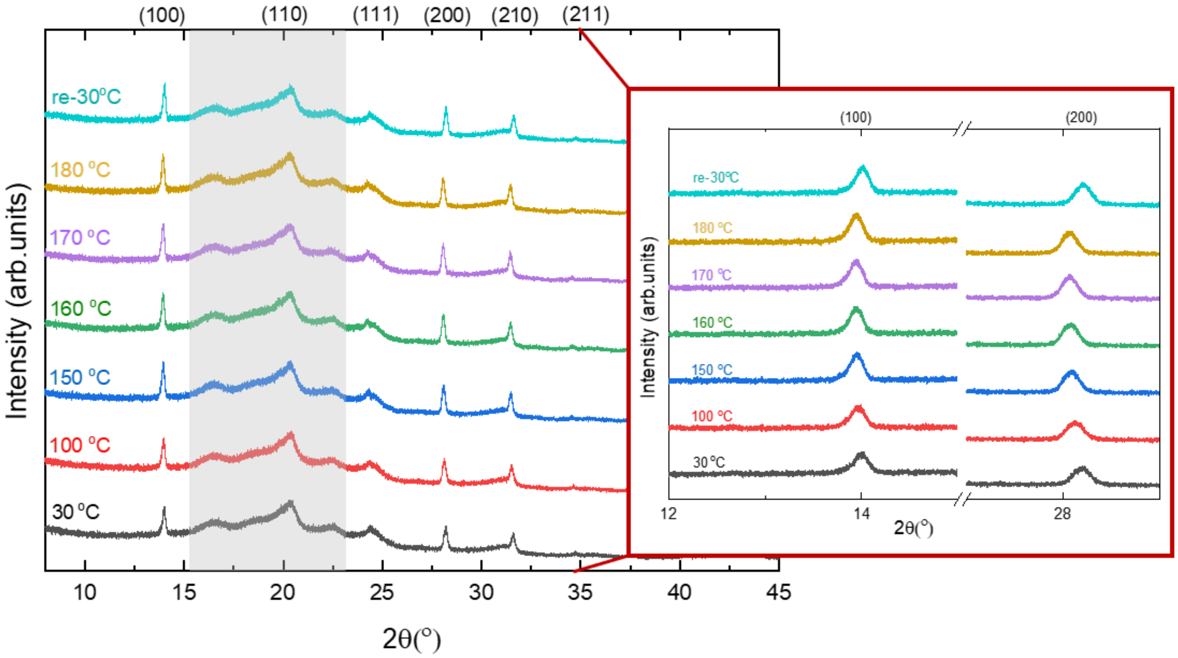

Figure S13: Temperature dependence XRD analysis of the $MA_{0.55}FA_{0.45}PbI_3$ pellet.

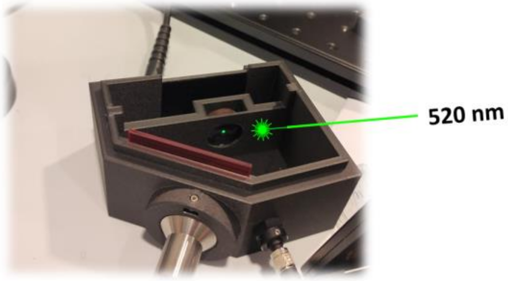

Figure S14: Photoluminescence (PL) set up to measure the MHP pellets. Consisting of an optical fiber, a filter, a spectrophotometer, and a pellet holder facing the laser 520 nm laser. For thin films, the holder is exchanged based on the sample holder's dimensions.

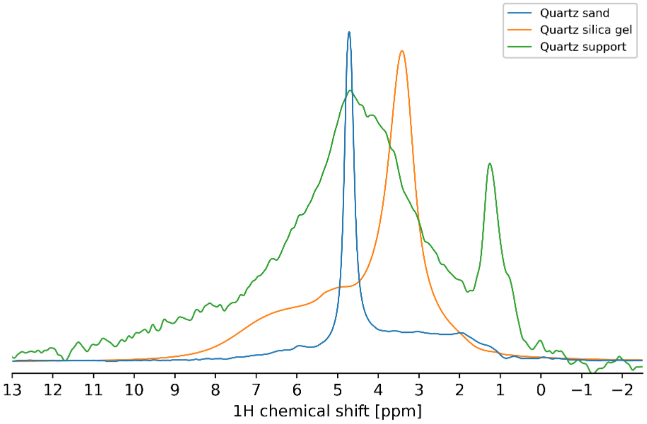

Figure S15: ^{1}H MAS NMR spectra of quartz sand (in blue), quartz silica gel (in orange), and quartz support used in this study (in green), acquired at B_0 = 19.97 T (ν_0= 850.13 MHz for ^{1}H) and ν_R= 25 kHz, 1.6 mm rotor, 64 scans, D1= 55s.

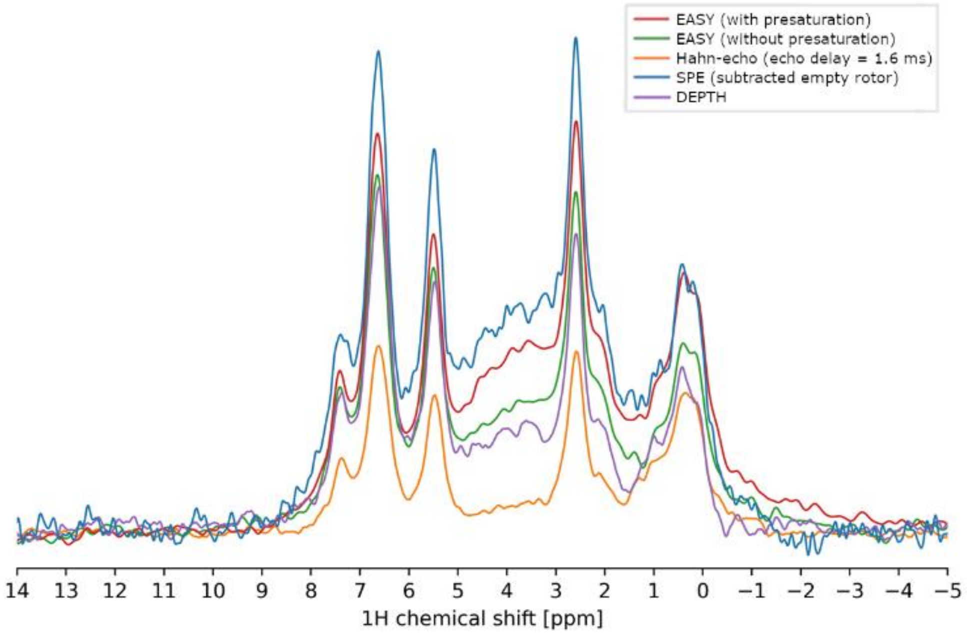

Figure S16: ^{1}H MAS NMR spectra of the $MA_{45}FA_{55}PbI_3$ thin film with substrate, showing the effect different pulse sequences meant to suppress unwanted signals. Scaled to identical intensity per scan. Acquired at B_0 = 19.97 T (ν_0= 850.13 MHz for ^{1}H) and ν_R= 25 kHz. The EASY and Hahn-echo sequences used 256 scans, the onepulse and the DEPTH 64 scans.

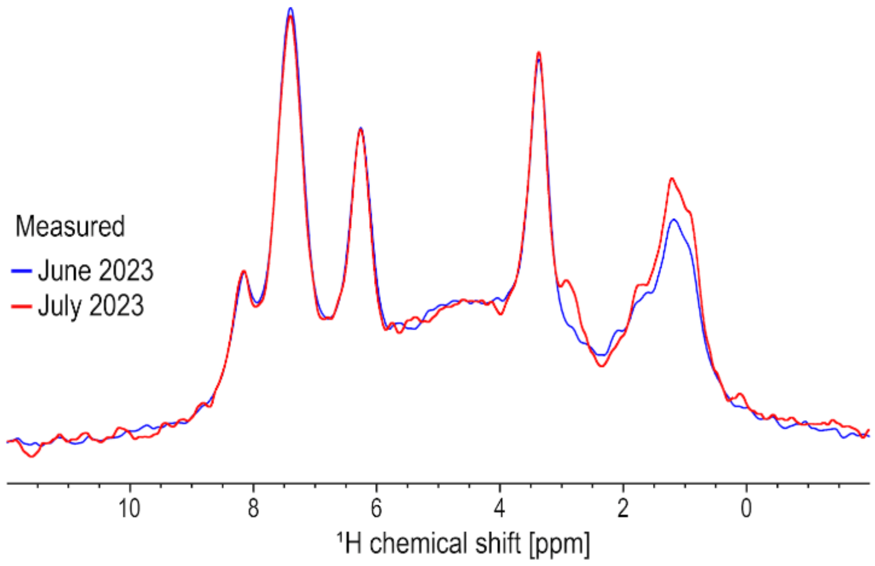

Figure S17: ^{1}H MAS NMR DEPTH spectra of the $MA_{45}FA_{55}PbI_3$ thin film with substrate measured at separate dates, demonstrating minor sample instability. Acquired at B_0 = 19.97 T (ν_0= 850.13 MHz for ^{1}H) and ν_R= 25 kHz, 1.6mm rotor, 64 scans.

Table S1: Longest T_1values determined in the MAS NMR experiments of the four mixed $MA_{1-x}FA_xPbI_3$ samples and the thin film. ^1H T_1s were determined using the saturation-recovery sequence, ^{13}C T_1s were determined using the inversion recovery sequence. Note that the thin film was measured at B_0 = 19.97 T (ν_0= 850.13 MHz for ^1H) while the pressed powders were measured at B_0 = 14.09 T (ν_0= 600.13 MHz for ^1H).

**Sample**	**T_1 (s)**	
	**^1H**	**^{13}C**
$MA_{0.75}FA_{0.25}PbI_3$	26	53
$MA_{0.63}FA_{0.37}PbI_3$	20	46
$MA_{0.50}FA_{0.50}PbI_3$	20	46
$MA_{0.25}FA_{0.75}PbI_3$	17	35
$MA_{0.45}FA_{0.55}PbI_3$ thin film	11	

Table S2: Quantification of FA/MA ratios using the direct ^{13}C MAS NMR and the ^1H MAS NMR spectra of the four mixed $MA_{1-x}FA_xPbI_3$ samples and the thin film.

**Sample**	**MA^+: FA^+ ratios**			
	**^1H SPE (%)***		**^{13}C SPE (%)†**	
	**MA^+**	**FA^+**	**MA^+**	**FA^+**
$MA_{0.75}FA_{0.25}PbI_3$	73.2 ± 2.0	26.8 ± 2.0	68.5 ± 4.2	31.5 ± 4.2
$MA_{0.63}FA_{0.37}PbI_3$	63.0 ± 2.0	37.0 ± 2.0	66.7 ± 0.1	33.3 ± 0.1
$MA_{0.50}FA_{0.50}PbI_3$	52.5 ± 2.7	47.5 ± 2.7	50.4 ± 0.8	49.6 ± 0.8
$MA_{0.25}FA_{0.75}PbI_3$	27.5 ± 1.8	72.5 ± 1.8	23.8 ± 0.7	76.7 ± 0.7
$MA_{0.45}FA_{0.55}PbI_3$ thin film	39.0 **	61.0 **		

*The ratios were obtained by fitting the signals for FA^+ and MA^+. The errors are the STD (standard deviation) of 4 different fits using DMFit and ssNAKE.

**Due to the convolution of non-perovskite signals in the spectrum, there is no sensible way to estimate a quantitative error margin for the thin film. We assume that the possible error margin for this sample is at least as large as those for the bulk compounds.

†The ratios were obtained by fitting the signals for FA^+ and MA^+. The errors are the STD (standard deviation) of a fit and an integration using DMFit and ssNAKE, however, the poor lineshape and limited fits make both ratios and errors unreliable compared to the ^1H results.

Table S3: Estimated time required to achieve an S/N of 100 in a ^1H MAS spectrum of a $MA_{45}FA_{55}PbI_3$ thin film on a substrate at different magnetic fields B_0, for various absorption layer-to-support ratios r. Changes in T_1 due to changes in the B_0 were not taken into account.

B_0 (^1H Larmor frequenc)	r = 0.008	r = 0.004	r = 0.002
400 MHz	28 h	112 h	446 h
850 MHz	2 h	8 h*	32 h
1200 MHz	0.6 h	2.4 h	9.5 h

*This work

Table S4: N atomic percentage by XPS-based and experimental cation ratio of stoichiometric pellets.

Sample	N atomic % from fitting using CasaXPS		ratio % obtained via XPS measurements	
	$C\text{-}NH_3$	$C\text{-}NH_2$	MA^+	FA^+
$MA_{75}FA_{25}PbI_3^*$	60.0 ± 0.21	40.0 ± 0.21	75.0 ± 0.3	25.0 ± 0.3
$MA_{63}FA_{37}PbI_3$	54.55 ± 0.19	45.45 ± 0.19	68.8 ± 0.2	31.2 ± 0.2
$MA_{50}FA_{50}PbI_3$	34.21 ± 0.21	65.79 ± 0.21	50.2 ± 0.3	49.8 ± 0.3
$MA_{25}FA_{75}PbI_3$	20.63 ± 0.20	79.37 ± 0.20	30.5 ± 0.3	69.5 ± 0.3

$C\text{-}NH_3$ signal integrated at ~ 402 eV and $C\text{-}NH_2$ signal integrated at ~ 400 eV.
*MA = 1N = 1x, FA = 2N = 2x; For $MA_{75}FA_{25}PbI_3$, 75x + 2x·25 = 100, x: 0.80

Table S5: Spectra features table of Pb 4f, I 3d, and C 1s core levels.

MA^+: FA^+ (angle)	Element/Transition	Peak width FWHM (eV)	Peak Energy (eV)
100 : 0	Pb $4f_{7/2}$	1.29	137.6
	Pb $4f_{5/2}$	1.29	142.4
	I $3d_{5/2}$	1.46	618.5
	I $3d_{3/2}$	1.46	630.0
	C 1s MA^+	1.67	286.0
75: 25	Pb $4f_{7/2}$	1.26	137.9
	Pb $4f_{5/2}$	1.26	142.7
	I $3d_{5/2}$	1.41	618.6
	I $3d_{3/2}$	1.41	630.1
	C 1s FA^+	1.57	288.3
	C 1s MA^+	1.57	286.3
63: 37	Pb $4f_{7/2}$	1.26	137.8
	Pb $4f_{5/2}$	1.26	142.6
	I $3d_{5/2}$	1.43	618.6
	I $3d_{3/2}$	1.43	630.1
	C 1s FA^+	1.61	288.0
	C 1s MA^+	1.61	286.2
50: 50	Pb $4f_{7/2}$	1.34	137.7
	Pb $4f_{5/2}$	1.34	142.5
	I $3d_{5/2}$	1.48	618.5
	I $3d_{3/2}$	1.48	629.9
	C 1s FA^+	1.60	288.1
	C 1s MA^+	1.60	286.2
25: 75	Pb $4f_{7/2}$	1.29	137.8
	Pb $4f_{5/2}$	1.29	142.7
	I $3d_{5/2}$	1.44	618.7
	I $3d_{3/2}$	1.44	630.2
	C 1s FA^+	1.53	288.0
	C 1s MA^+	1.53	286.2
0: 100 (310)	Pb $4f_{7/2}$	1.34	138.0
	Pb $4f_{5/2}$	1.34	142.8
	I $3d_{5/2}$	1.49	618.8
	I $3d_{3/2}$	1.49	630.3

	C 1s FA^{+}	1.70	288.1

Table S6: Estimation of the lattice parameters using X-ray diffraction data.

Sample (FA%)	Phase(s)	100 (002/110) 2θ °	200 (004/220) 2θ °	Lattice (Å)	FWHM 100 (002/110) 2θ °
0	Tetragonal*	(13.98/14.06)	(28.15/28.40)	6.30*	0.271 ± 0.003
25	(pseudo)Cubic	14.01	28.26	6.316	0.157 ± 0.001
37	Cubic	13.99	28.22	6.325	0.174 ± 0.001
50	Cubic	13.96	28.18	6.339	0.171 ± 0.002
75	Cubic	13.95	28.12	6.343	0.204 ± 0.002
100	Hexagonal/Cubic*	13.88	28.05	6.37*	-

Values taken from literature*

Table S7. Bandgap of halide perovskite pellets and additional information.

Sample	Phase(s)	Peak emission (eV)	FWHM (nm) Gaussian	FWHM (nm) Voigt
0% FA	Tetragonal	1.610	65.47 ± 0,36	54.52 ± 0,41
25%FA	(pseudo)Cubic	1.597	68.52 ± 0,42	56.96 ± 0,48
37%FA	Cubic	1.580	69.59 ± 0,42	56.95 ± 0,49
50%FA	Cubic	1.575	73.15 ± 0,35	62.13 ± 0,35
75%FA	Cubic	1.565	74.71 ± 0,32	69.63 ± 0,36
100%FA	Hexagonal/Cubic*	1.627	73.28 ± 0,18	71.85 ± 0,20

Values taken from literature*

Table S8-14. Stoichiometric Halide Perovskites Pellets additional information.

$MAPbI_3$	Precursors	MM [mg/ mmol]	n [mmol]	m [mg]	Measured [mg]	Pellet [mg]	NMR powder [mg]
	PbI_2	461.01	4.9	2258.95	2258.65	2004.3	1010
	MAI	158.97	4.9	778.95	778.90		
	m (total) [mg] Σ =			3037.90	3037.55		
	ZrO_2 Ø [mg]			30300			

$MA_{0.75}FA_{0.25}PbI_3$	Precursors	MM [mg/ mmol]	n [mmol]	m [mg]	Measured [mg]	Pellet [mg]	NMR powder [mg]
	PbI_2	461.01	4.9	2258.95	2258.50	2064.7	980
	MAI	158.97	4.9	584.21	584.84		
	FAI	171.97	4.9	210.66	210.88		
	m (total) [mg] Σ =			3053.83	3054.22		
	ZrO_2 Ø [mg]			30600			

$MA_{0.63}FA_{0.37}PbI_3$	Precursors	MM [mg/ mmol]	n [mmol]	m [mg]	Measured [mg]	Pellet [mg]	NMR powder [mg]
	PbI_2	461.01	4.9	2258.95	2258.63		

	MAI	158.97	4.9	490.74	490.73		
	FAI	171.97	4.9	311.780	312.00		
	m (total) [mg] Σ =			3061.47	3061.36	2013.9	1001
	ZrO_2 Ø [mg]			30600			

$MA_{0.50}$ $FA_{0.50}$ PbI_3	Precursors	MM [mg/ mmol]	n [mmol]	m [mg]	Measured [mg]	Pellet [mg]	NMR powder [mg]
	PbI_2	461.01	4.9	2258.95	2258.87		
	MAI	158.97	4.9	389.48	389.56		
	FAI	171.97	4.9	421.32	421.90		
	m (total) [mg] Σ =			3069.75	3070.33	2040.1	1012
	ZrO_2 Ø [mg]			30700			

$MA_{0.25}$ $FA_{0.75}$ PbI_3	Precursors	MM [mg/ mmol]	n [mmol]	m [mg]	Measured [mg]	Pellet [mg]	NMR powder [mg]
	PbI_2	461.01	4.9	2258.95	2258.6		
	MAI	158.97	4.9	194.74	195.74		
	FAI	171.97	4.9	631.99	632.82		
	m (total) [mg] Σ =			3085.67	3087.16	2073.6	1002
	ZrO_2 Ø [mg]			30600			

$FAPbI_3$	Precursors	MM [mg/ mmol]	n [mmol]	m [mg]	Measured [mg]	Pellet [mg]	NMR powder [mg]
	PbI_2	461.01	4.9	2258.95	2258.81		
	FAI	171.97	4.9	842.65	842.55		
	m (total) [mg] Σ =			3101.60	3101.36		
	ZrO_2 Ø [mg]			30500		2014.9	1015

Table S15: Pellets density estimation after 30min at 5 metric tons (~158.66 MPa)

Target ID	mass of target [g]	thickness [cm]	diameter [cm]	volume [cm^3]	density [g/cm^3]
$MAPbI_3$	1999.05	0.164	2	0.515	3879.99
$MA_{0.75}FA_{0.25}PbI_3$	2055.96	0.164	2	0.515	3990.44
$MA_{0.63}FA_{0.37}PbI_3$	2010.15	0.161	2	0.506	3974.23
$MA_{0.50}FA_{0.50}PbI_3$	2034.32	0.162	2	0.509	3997.19
$MA_{0.25}FA_{0.75}PbI_3$	2063.73	0.161	2	0.506	4080.16
$FAPbI_3$	2007.52	0.161	2	0.506	3969.03
